# Identification of a novel type III secretion-associated outer membrane-bound protein from *Xanthomonas campestris* pv. *campestris*

**DOI:** 10.1038/srep42724

**Published:** 2017-02-15

**Authors:** Lei Li, Rui-Fang Li, Zhen-Hua Ming, Guang-Tao Lu, Ji-Liang Tang

**Affiliations:** 1 State Key Laboratory for Conservation and Utilization of Subtropical Agro-bioresources, College of Life Science and Technology, Guangxi University, 100 Daxue Road, Nanning, Guangxi 530004, China; 2 Guangxi Key Laboratory of Biology for Crop Diseases and Insect Pests, Plant Protection Research Institute, Guangxi Academy of Agricultural Sciences, 174 Daxue Road, Nanning, Guangxi 530007, China

## Abstract

Many bacterial pathogens employ the type III secretion system (T3SS) to translocate effector proteins into eukaryotic cells to overcome host defenses. To date, most of our knowledge about the T3SS molecular architecture comes from the studies on animal pathogens. In plant pathogens, nine Hrc proteins are believed to be structural components of the T3SS, of which HrcC and HrcJ form the outer and inner rings of the T3SS, respectively. Here, we demonstrated that a novel outer membrane-bound protein (HpaM) of *Xanthomonas campestris* pv. *campestris* is critical for the type III secretion and is structurally and functionally conserved in phytopathogenic *Xanthomonas* spp. We showed that the C-terminus of HpaM extends into the periplasm to interact physically with HrcJ and the middle part of HpaM interacts physically with HrcC. It is clear that the outer and inner rings compose the main basal body of the T3SS apparatus in animal pathogens. Therefore, we presume that HpaM may act as a T3SS structural component, or play a role in assisting assembling or affecting the stability of the T3SS apparatus. HpaM is a highly prevalent and specific protein in *Xanthomonas* spp., suggesting that the T3SS of *Xanthomonas* is distinctive in some aspects from other pathogens.

Many Gram-negative bacterial pathogens of plants and animals employ the type III secretion system (T3SS) to deliver effector proteins into host cells, where they manipulate host cellular pathways to benefit the pathogens and thus allow the bacteria to successfully multiply. The T3SS apparatus is a complex macromolecular nanomachine that is composed of more than 20 proteins^1^,^2^,^3^,^4^. A typical T3SS apparatus consists of three parts: an extracellular pilus-like (plant pathogens) or needle-like (animal pathogens) appendage, a membrane-spanning basal body and the peripheral inner membrane cytoplasmic components. The basal body supports the pilus or needle appendage by anchoring the appendage on the bacterial membranes. Normally, the T3SS needle from animal pathogens is about 40–80 nm in length and the pilus from plant pathogens is up to 2 μm. The basal body is built of stacked toroids: an outer membrane ring extends to the periplasm and associates with the inner membrane ring. The cytoplasmic components are the ATPase complex and predicted cytoplasmic ring (C-ring)^5^,^6^,^7^,^8^. To date, most of our knowledge about the T3SS molecular architecture comes from the studies on animal pathogens such as *Shigella, Salmonella*, and *Yersinia*. The T3SS of plant pathogenic bacteria is encoded by a cluster of more than 20 *hrp* (hypersensitive response and pathogenicity) genes. Inactivation of the T3SS abolished the ability of the pathogens to produce disease lesions in host plants and to elicit hypersensitive response (HR) in nonhost or resistant plants. Comparative sequence analyses revealed that nine *hrp* genes (termed *hrc* for *hrp* conserved) are conserved among different plant pathogens and the Hrc proteins are highly homologous to the proteins constituting the T3SS apparatus of animal pathogens. In addition, several studies have shown that the T3SS of plant pathogens can secrete effector proteins from animal pathogens and plant pathogen effectors can be secreted by the T3SS of animal pathogens^9^,^10^. Based on these facts, it is presumed that the Hrc proteins are the components of the T3SS in plant pathogens and the core T3SS apparatus may be conserved among plant and animal pathogens^6^,^11^. According to their homology to the T3SS components of animal pathogens, the function of the nine conserved Hrc proteins is believed to be: (1) HrcC is an outer membrane ring protein; (2) HrcJ is an inner membrane ring protein; (3) HrcR, S, T and U are integral inner membrane proteins with periplasmic extensions, taking part in the rod formation of the T3SS apparatus; and (4) HrcV, Q and N are inner membrane or peripheral cytoplasmic proteins engaged in initiation of effector secretion from the cytoplasm^6^,^8^,^11^,^12^,^13^,^14^.

*Xanthomonas* is a large genus of Gram-negative bacteria, which comprises 27 species and some of which include multiple pathovars. Many members of the genus are important plant pathogens, such as *X. campestris* pv. *campestris* (the crucifer black rot pathogen), *X. citri* subsp. *citri* (the citrus canker pathogen), *X. euvesicatoria* (the pepper and tomato bacterial spot pathogen), *X. oryzae* pv. *oryzae* (the rice bacterial blight pathogen), and *X. oryzae* pv. *oryzicola* (the rice bacterial leaf streak pathogen), and most of which rely on an efficient T3SS for their pathogenicity^15^,^16^. The T3SS-encoding *hrp* cluster of *Xanthomonas* spp. consists of six operons (*hrpA* to *hrpF*) which harbor more than 20 different genes including the nine conserved *hrc* genes^17^,^18^,^19^. Recently, we identified a novel outer membrane-bound protein that is involved in the HR and pathogenicity of *X. campestris* pv. *campestris (Xcc*), which was designated as HpaM (for Hrp-associated membrane-bound protein). Here, we present evidences showing that the protein is essential for type III secretion and conserved in *Xanthomonas* spp.

## Results

### HpaM is essential for the virulence and HR induction of *Xcc*

In our previous work, we isolated a large number of *Xcc* mutants from a library constructed by the transposon Tn5*gusA*5 insertion in the genome of *Xcc* wild-type strain 8004. One of the mutants, 083E12, was due to a Tn5*gusA*5 insertion in the ORF *XC*_*2847* (named *hpaM* in this study). Plant tests showed that the mutant strain 083E12 almost completely lost virulence and hardly induced any disease or HR symptoms in the host plant Chinese radish or the non-host plant pepper (cultivar ECW-10R). The gene *XC*_*2847* was annotated to be 1161 bp in length, locating at nucleotide (nt) positions from the 3426325^th^ to the 3427485^th^ nt, and predicted to encode a hypothetical protein^20^. Using a standard 5′-RACE method, the transcription initiation site (TIS) of *XC*_*2847* was mapped at 89 nucleotides downstream of the predicted translational start codon GTG (Fig. S1). There is an in-frame ATG codon 22 bp downstream of the determined TIS (Fig. S1). Based on these data, we propose that the *XC*_*2847* ORF should start with the ATG and consist of 1050 bp instead of 1161 bp.

**Figure 1:**
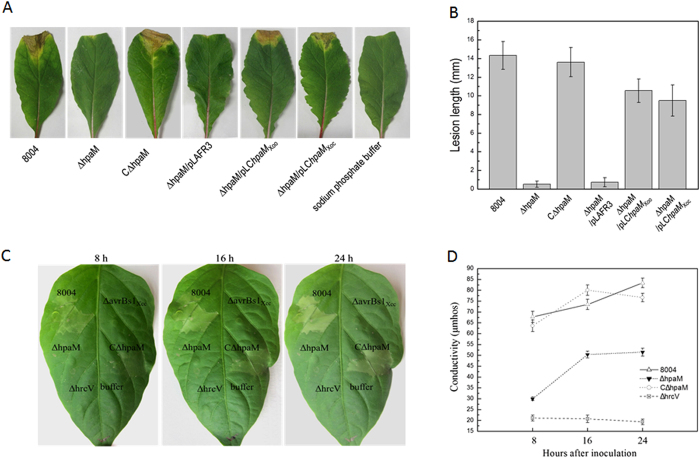
HpaM is essential for pathogenicity and HR induction of *Xcc*. The *Xcc* wild-type strain 8004 and its derivatives from overnight culture were washed and resuspended in 10 mM SPB or sterile distilled water (for electrolyte leakage assay) to an OD_600_ of 0.1 (1 × 10^8^ CFU ml^−1^). (**A**) Disease symptoms on Chinese radish (*Raphanus sativus*) leaves. *Xcc* strains were inoculated by cutting leaves with scissors dipped in the bacterial suspensions. (**B**) Lesion lengths were scored 10 days postinoculation. Values represent means and standard deviation from twenty inoculated leaves in one experiment. The experiment was repeated three times with similar results. (**C**) HR symptoms induced in pepper leaves (*Capsicum annuum* cv. ECW-10R) by *Xcc* strains. Approximately 5 μl bacterial resuspension (1 × 10^8^ CFU ml^−1^) was infiltrated into the leaf mesophyll tissue with a blunt-end plastic syringe. Pictures were taken at 8, 16 and 24 h after infiltration. Three replications were done in each experiment, and the experiment was repeated three times. The results presented are from a representative experiment, and similar results were obtained in all other independent experiments. *hrcV* and *avrBs1*_*Xcc*_ deletion mutants ΔhrcV and ΔavrBs1_Xcc_ were used as negative controls. (**D**) Electrolyte leakage from pepper leaves inoculated with *Xcc* strains. For each sample, four 0.4 cm^2^ leaf disks were collected from the infiltrated area and incubated in 5 ml distilled water. Conductivity was measured with a DDS-307A conductometer. Three samples were taken for each measurement in each experiment. Results presented are from a representative experiment, and similar results were obtained in two other independent experiments. *hrcV* deletion mutant ΔhrcV was used as a negative control.

To facilitate further studies on the function of *hpaM*, a deletion mutant, named ΔhpaM, was constructed by using the suicide vector pK18mob*sacB* (Table S1). Simultaneously, a complemented strain was also constructed by introducing the recombinant plasmid pLC*hpaM*, which carries an entire *hpaM* gene, into the mutant ΔhpaM. The resulting complemented strain was named as CΔhpaM (Table S1). As anticipated, the mutant ΔhpaM could hardly induce visible disease or HR symptoms (Fig. 1). However, the complemented strain CΔhpaM could produce wild-type disease and HR symptoms (Fig. 1), suggesting that the pathogenicity and HR of ΔhpaM could be restored by *hpaM in trans*. The growth in planta of the *hpaM* mutant was suppressed significantly, although its growth rate was not affected in minimal medium (Fig. S2), suggesting that mutation in *hpaM* decreased significantly fitness in planta. Taken together, the above data indicate that HpaM is essential for the virulence and HR induction of *Xcc*.

### HpaM is required for T3Es secretion of *Xcc*

As mentioned above, the T3SS is critical for the pathogenicity and HR induction of *Xcc*. To gain an insight into the mechanisms by which HpaM affects the virulence and HR induction, we examined whether HpaM is involved in the T3SS. The T3SS of *Xcc* is encoded by six *hrp* operons (*hrpA* to *hrpF*) and the expression of the *hrp* operons is positively controlled by several key regulators including HrpG and HrpX^21^,^22^,^23^. To determine whether HpaM influences the expression of *hrp* genes, the plasmid-driven promoterless β-glucuronidase (*gusA*) transcriptional fusion reporters of *hrpG* and *hrpX* regulators as well as the six *hrp* operons, in which a DNA fragment containing the promoter region of each of the *hrp* operons (*hrpA* to *hrpF*) and *hrpG* and *hrpX* genes fused to the promoterless *gusA* gene with its ribosome binding site (RBS) was cloned into the vector pLAFR6 (Table S1), were introduced from *E. coli* JM109 by triparental conjugation into the *hpaM* mutant ΔhpaM and the wild-type strain 8004, and transconjugants (reporter strains) were screened on NYG medium as described previously^22^. As the expression of the *hrp* genes is induced in minimal media but inhibited in rich media^23^, β-glucuronidase (GUS) activities produced by the obtained reporter strains (Table S1) were measured after cultivation in MMX minimal medium. The results revealed that each of the reporters produced similar GUS activity in wild-type and *hpaM* deletion backgrounds (Fig. S3A), suggesting that mutation of *hpaM* did not affect the expression of the *hrp* genes. To clarify whether the expression of *hpaM* is subject to HrpG and HrpX regulation, the promoter-*gusA* transcriptional fusion reporter of *hpaM* was constructed. A 404-bp DNA fragment upstream of the *hpaM* ORF, amplified from the wild-type strain 8004, was fused with the coding region of promoterless *gusA* gene and cloned into pLAFR6, generating the reporter plasmid named pGUS*hpaM* (Table S1). The GUS activities produced by the reporter plasmid in wild-type background and *hrpG* or *hrpX* mutation background were not significantly different (P = 0.05 by *t* test) (Fig. S3B), indicating that the expression of *hpaM* is not controlled by HrpG and HrpX. In addition, the reporter plasmid pGUS*hpaM* in *hpaM* mutation background and wild-type background produced similar GUS activities (Fig. S3B), implying that HpaM plays no impact on its own expression.

**Figure 2:**
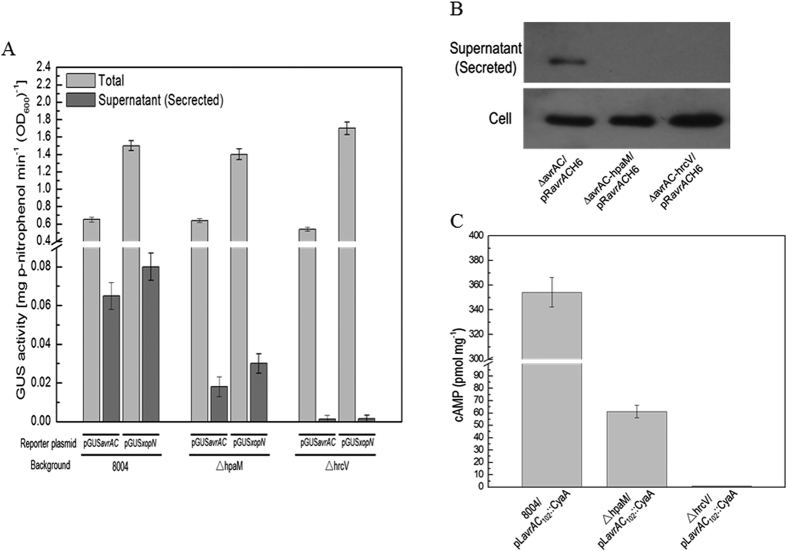
HpaM is essential for secretion of T3SS effectors in *Xcc*. Type III secretion signal sequence-*gusA* fusion reporter plasmids pGUS*avrAC* and pGUS*xopN* were introduced into *Xcc* strains. The resulting recombinant strains were cultured in XVM2 medium for 12 h and the *β*-glucuronidase (GUS) activities were determined. Values are the means ± standard deviation from three repeats. (**A**) GUS activities in the cultural supernatant (Secreted) and the total culture (Total) produced by pGUS*avrAC* and pGUS*xopN* in different background strains. (**B**) Western blot assay. The recombinant plasmid pR*avrAC*H6, which contains the T3E AvrAC encoding sequence fused with 6×His tag in its C-terminus, was introduced into *Xcc* strains. The resulting recombinant strains were cultured in XVM2 medium for 12 h and proteins in cultural supernatant (secreted protein) were collected by ultra-filtration using Amicon Ultra-15 centrifugal filter (Millipore Corporation, Billerica, MA, USA) and the total proteins in *Xcc* cells were prepared as previously described^62^. 30 μg of secreted or cell protein was electrophoresed in SDS-PAGE gel and transferred to a PVDF membrane. The presence of AvrAC was detected by anti-His_6_ monoclonal antibody. (**C**) Cya protein translocation assay. The pL*avrAC*_102_::CyaA fusion construct was transferred into *Xcc* strains and the resulting recombinant strains were then used to inoculate Chinese radish (*Raphanus sativus*) leaves. The cAMP level was determined 24 h post-inoculation. Values given are the means ± standard deviations of triplicate measurements from a representative experiment; similar results were obtained in two other independent experiments. 8004, wild type strain; ∆hpaM, *hpaM* deletion mutant; ∆hrcV, *hrcV* deletion mutant.

We further investigated whether HpaM is involved in T3Es secretion. It is well known that T3Es have a modular structure and the targeting signal generally resides in the N-terminal 50 or 100 amino acids (aa)^24^. Two reporter plasmids, pGUS*avrAC* and pGUS*xopN* (Table S1), were employed to study the secretion efficiency of *Xcc* T3SS. The reporters were previously constructed by fusing the promoterless *gusA* gene with a fragment including the promoter and targeting signal-encoding region of *avrAC (XC*_*1553*) or *xopN (XC*_*0241*), which encode the T3Es AvrAC and XopN, respectively^25^,^26^. pGUS*avrAC* and pGUS*xopN* were introduced into the *hpaM* mutant strain ΔhpaM and the wild-type strain 8004, respectively. The plasmids were also introduced into the *hrcV*-deficient mutant strain ΔhrcV as negative controls. HrcV is a conserved inner membrane protein of the core T3SS and the mutant ΔhrcV is defective in type III secretion^13^. The recombinant plasmid pL6*gus*, which was constructed by cloning a 1,832-bp promoterless *gusA* ORF into the promoterless cloning site of the plasmid pLAFR6, was introduced into the wild-type strain 8004 and the resulting strain 8004/pL6*gus*, which did not produced any significant GUS activity, was used as a negative control for the GUS assay. As shown in Fig. 2A,B, both reporters produced large amount of GUS activity in the wild-type and *hpaM* mutation backgrounds; however, the GUS activities in the cultural supernatants of the *hpaM* mutation background strains were significantly lower than those in the cultural supernatants of the wild-type background strains (*P* = 0.01 by *t* test), implying that mutation of *hpaM* significantly diminished the secretion of the T3Es AvrAC and XopN.

To further verify the effect of *hpaM* on the type III secretion, western blot assay was performed to examine the secretion of the T3E AvrAC in the *hpaM* mutation background. For this purpose, an *avrAC* deletion mutant (∆avrAC) and an *avrAC/hpaM* double deletion mutant (∆avrAC-hpaM) were constructed. Another double deletion mutant (∆avrAC-hrcV) that lacked *avrAC* and *hrcV* was also constructed and used as a negative control strain. The recombinant plasmid pR*avrAC*H6, which was constructed by fusing 6×His-tag coding sequence to the 3′ end of the *avrAC* gene with its own promoter and cloning the fused fragment into the promoterless cloning site of the plasmid pLAFR6, was then introduced into the mutants. The resulting strains ∆avrAC/pR*avrAC*H6, ∆avrAC-hpaM/pR*avrAC*H6 and ∆avrAC-hrcV/pR*avrAC*H6 (Table S1) were used to test the secretion of AvrAC protein by western blot assay. As shown in Fig. 2B, AvrAC protein was present in the cells of all the strains tested and the cultural supernatant of the strain ∆avrAC/pR*avrAC*H6. Similar to the negative control strain ∆avrAC-hrcV/pR*avrAC*H6, no AvrAC protein was detected in the cultural supernatant of the strain ∆avrAC-hpaM/pR*avrAC*H6 under the test conditions (Fig. 2B), indicating that deletion of *hpaM* abolished the secretion of AvrAC. These data confirm that HpaM is indispensable for the type III secretion of *Xcc*.

To further estimate the effect of HpaM on T3Es translocation into plant cells, the N-terminal 102 aa of the T3E AvrAC were fused with the calmodulin-dependent reporter protein Cya^27^ and the resulting reporter plasmid, named pL*avrAC*_102_::CyaA (Table S1), was introduced into the *hpaM* mutant strain ΔhpaM, the wild-type strain 8004, and the T3SS-defective *hrcV* mutant ΔhrcV. The obtained recombinant strains were inoculated into radish leaves at 10^8^ cfu ml^−1^ (OD_600_ = 0.1), and the cAMP levels were measured 24 h post-inoculation. Strain ΔhrcV/pLAFR6, which was constructed by introducing the vector pLAFR6 into the *hrcV* mutant strain ΔhrcV, was used as a negative control. As shown in Fig. 2C, the cAMP level in the leaves inoculated with the wild-type strain harboring the reporter plasmid was higher than that in the leaves inoculated with the mutants carrying the reporter plasmid. As the Cya protein produces a measurable cAMP level only in plant cells but not in bacterial cells or plant apoplasts^28^, the result reveals that HpaM is essential for T3Es translocation into plant cells.

*Xcc* secretes a series of extracellular enzymes including exoproteases by the type II secretion system (T2SS). To evaluate whether HpaM affects the T2SS, we compared the exoprotease activities produced by the *hpaM* mutant strain ΔhpaM and the wild type strain 8004. The result showed that the two strains produced similar enzyme activities (Fig. S4A), suggesting that HpaM is not involved in the T2SS. The extracellular polysaccharide (EPS) production and the motility of the mutant ΔhpaM were also determined. No significant difference on either EPS production or motility was observed between the mutant and the wild type (Fig. S4), indicating that HpaM does not affect EPS production and cell motility.

### HpaM is located in the outer membrane of *Xcc*

The HpaM protein of *Xcc* consists of 349 aa. Domain analysis with the SMART (Simple Modular Architecture Research Tool) program (http://smart.embl-heidelberg.de) showed that HpaM contains a signal peptide (residues1–22), and 6 PbH1 domains (residues 120–163, 180–202, 203–225, 226–248, 249–271, and 288–311) which were annotated as “parallel β-helix repeats”. A prediction by the TMPRED program (http://www.ch.embnet.org/software/TMPRED_form.html) revealed that the residues from the 8^th^ to the 29^th^ aa in the N-terminal domain of HpaM constitute transmembrane helices (total score: 1405). These suggest that HpaM may be a membrane-bound protein.

**Figure 3:**
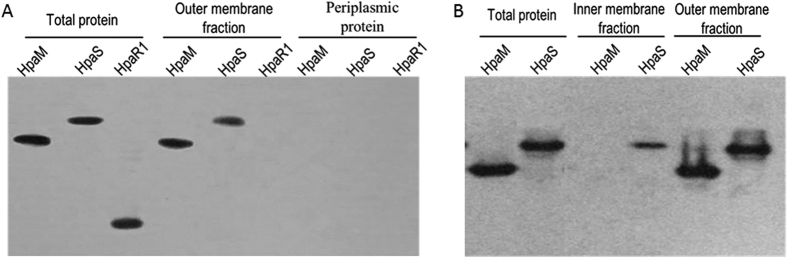
Subcellular localization of HpaM by western blot analysis. *Xcc* strains were cultured to an OD_600_ of 1.0 and proteins were prepared using the method described by Feilmeier and associates (2000) (**A**) or the method described by Chen and associates (2010) (**B**). 30 (for total protein) or 10 μg of protein sample was separated by SDS-PAGE electrophoresis and transferred to a PVDF membrane. The presence of HpaM was detected by anti-His_6_ monoclonal antibody. The histidine sensor kinase HpaS and the transcription regulator HpaR1 were used as controls. HpaM, protein sample was prepared from strain ΔhpaM/pR*hpaM*H6; HpaS, protein sample was prepared from strain ∆hpaS/pR*hpaS*H6; HpaR1, protein sample was prepared from strain ∆hpaR1/pR*hpaR1*H6.

To validate whether HpaM is a membrane-bound protein, the cellular location of HpaM in *Xcc* was determined. We constructed a recombinant strain, ΔhpaM/pR*hpaM*H6, which expressed HpaM with a 6×His tag on its C-terminus in the *hpaM* deletion strain ΔhpaM. The total, periplasmic, and outer membrane protein fractions of the strain ΔhpaM/pR*hpaM*H6 grown at the late log phase were prepared. Western blot analysis revealed HpaM present in the total-protein and the outer membrane fractions but not in the periplasmic protein fraction (Fig. 3A). The cytoplasm protein HpaR1^29^ and the outer and inner membrane protein HpaS^21^ were taken as controls (Fig. 3A). To further determine whether HpaM also locates in the inner membrane, the outer and inner membrane fraction proteins were prepared using the method as described by Chen and associates^30^. The result showed that HpaM was detected only in the outer membrane fraction but not in the inner membrane fraction, while the control protein HpaS was detected in both outer and inner membrane fractions (Fig. 3B). These combined data indicate that HpaM is an outer membrane protein in *Xcc*.

### HpaM physically interacts with HrcC and HrcJ

**Figure 4:**
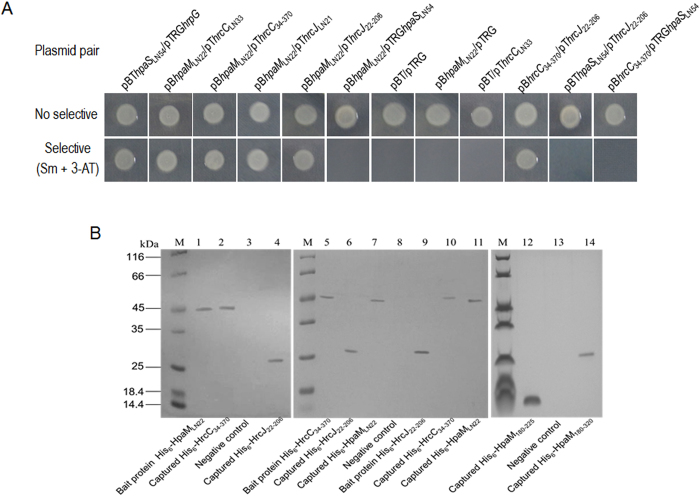
HpaM interacts with HrcC and HrcJ. (**A**) Bacterial two-hybrid assays. The BacterioMatch II two-hybrid system was used to test the interaction between HpaM and HrcC or HrcJ. The reporter strain XL1-Blue MRF′ harboring different plasmid pairs was grown on no selective plates and double-selective indicator plates containing 5 mM 3-amino-1,2,4-triazole (3-AT) and 12.5 μg ml^−1^ streptomycin, respectively. Protein-protein interaction activated the expression of the genes *HIS3* and *addA* in the reporter strain, resulting in resistance to 3-AT and streptomycin. (**B**) Pull-down assays. His_6_-tagged fusion proteins were overexpressed and purified. Streptavidin sepharose beads were used to immobilize biotinylated His_6_-HpaM_LN22_, His_6_-HrcC_34–370_ or His_6_-HrcJ_22–206_, the potential prey protein was mixed with the bait protein and incubated. After elution, samples were separated on 12% SDS-PAGE and visualized by coomassie blue staining. Lanes: 1, biotinylated bait protein His_6_-HpaM_LN22_; 2, pull-down of His_6_-HrcC_34–370_ by His_6_-HpaM_LN22_; 3, bait protein His_6_-HpaM_LN22_ mixed with protein His_6_-HpaR1(negative control); 4, pull-down of protein His_6_-HrcJ_22–206_ by His_6_-HpaM_LN22_; 5, biotinylated bait protein His_6_-HrcC_34–370_; 6, pull-down of protein His_6_-HrcJ_22–206_ by His_6_-HrcC_34–370_; 7, pull-down of protein His_6_-HpaM_LN22_ by His_6_-HrcC_34–370_; 8, bait protein His_6_-HrcC_34–370_ mixed with protein His_6_-HpaR1(negative control); 9, biotinylated bait protein His_6_-HrcJ_22–206_; 10, pull-down of protein His_6_-HrcC_34–370_ by His_6_-HrcJ_22–206_; 11, pull-down of protein His_6_-HpaM_LN22_ by His_6_-HrcJ_22–206_; 12, pull-down of truncated protein His_6_-HpaM_LN180–225_ by His_6_-HrcC_34–370_; 13, biotinylated His_6_-HrcC_34–370_ was mixed with protein His_6_-HpaR1 (negative control); 14, pull-down of truncated protein His_6_-HpaM_LN180–320_ by His_6_-HrcJ_22–206_; M, molecular mass marker.

The above data demonstrate that HpaM locates in the bacterial outer membrane and contributes to T3Es secretion, but is not involved in the regulation of the T3SS expression. From these facts we presumed that HpaM may act as a component of T3SS apparatus or a factor affecting the assembly or stability of the T3SS apparatus. To verify these possibilities, we employed the BacterioMatch II two-hybrid system (Stratagene, La Jolla, CA, USA) to determine whether HpaM physically interacts with the T3SS apparatus outer and inner membrane ring proteins HrcC and HrcJ^31^. A truncated *hpaM* gene excluding the N-terminal 22-aa signal peptide coding sequence was cloned into the bait vector pBT, yielding a recombinant plasmid named pB*hpaM*_LN22_ (Table S1). DNA fragments of truncated *hrcC* and *hrcJ* (excluding the N-terminal 33- and 21-aa signal peptide encoding sequences of *hrcC* and *hrcJ*, respectively) were fused into the target vector pTRG, yielding recombinant plasmids named pT*hrcC*_LN33_ and pT*hrcJ*_LN21_ (Table S1). The plasmids were introduced into the reporter strain XL1-Blue MRF′. The resulting recombinant strains, which harbor a pair of plasmids (Table S1) were tested for their growth ability on the double-selective indicator plate. In the reporter strain, if the HpaM and HrcC or HrcJ proteins interact with each other, the expression of *HIS3* and *addA* reporter genes will be activated, leading to the growth of the bacterial cells in the presence of 3-amino-1, 2, 4-triazole (3-AT) and streptomycin; however, if no interaction between the proteins occurs, the bacteria cannot grow in the same conditions. As shown in Fig. 4A, like the positive control strain XL1-Blue MRF′/pBT*hpaS*_LN54_/pTRG*hrpG* that showed an interaction between the histidine kinase HpaS and the response regulator HrpG of a two-component regulatory system^21^, the reporter strain XL1-Blue MRF′ harboring the plasmid pair pB*hpaM*_LN22_/pT*hrcC*_LN33_ or pB*hpaM*_LN22_/pT*hrcJ*_LN21_ grew well in the selective agar plate, while the negative control strains (the reporter strain harboring the plasmid pair pBT/pTRG, pB*hpaM*_LN22_/pTRG, or pBT/pT*hrcC*_LN33_) did not grow (Fig. 4A). These results indicate that HpaM interacts with HrcC as well as HrcJ in the reporter strain XL1-Blue MRF′. To evaluate whether the interaction between HpaM and HrcC or HrcJ is specific, the membrane-bound protein HpaS was included in the bacterial two-hybrid analysis. A truncated HpaS protein (lacking the N-terminal 54 aa transmembrane domain encoding sequence) was cloned into the target vector pTRG and the obtained plasmid pTRG*hpaS*_LN54_ was used in the analysis. The result showed that the reporter strain XL1-Blue MRF′ harboring the plasmid pair pB*hpaM*_LN22_/pTRG*hpaS*_LN54_ could not grow on the selective agar plate, indicating no interaction existed between HpaM and HpaS (Fig. 4A). It has been supposed that the periplasmic domains of the HrcC and HrcJ proteins interact with each other and compose the T3SS periplasmic rod of the T3SS apparatus^32^. We therefore tested whether HpaM interacts with the periplasmic domains of HrcC and HrcJ. For this purpose, a 1011 bp DNA fragment encoding the aa from the 34^th^ to the 370^th^ of HrcC and a 555-bp fragment encoding the aa from the 22^th^ to the 206^th^ of HrcJ were amplified and cloned into the target vector pTRG, yielding recombinant plasmids named pT*hrcC*_34–370_ and pT*hrcJ*_22–206_ (Table S1). As shown in Fig. 4A, the reporter strain XL1-Blue MRF′ harboring the plasmid pair pB*hpaM*_LN22_/pT*hrcC*_34–370_ or pB*hpaM*_LN22_/pT*hrcJ*_22–206_ was able to grow on the selective agar plate, indicating that HpaM interacts with the periplasmic domains of HrcC and HrcJ in the reporter strain.

To confirm the interactions, pull-down biotinylated protein-protein assays were performed. For this purpose, an attempt was made to overproduce recombinant 6×His-tagged truncated HrcC and HrcJ proteins by cloning truncated *hrcC* and *hrcJ* excluding the N-terminal 33- and 21-aa signal peptide encoding sequences into the expression vector pET-30a. However, we failed to obtain soluble form of the fusion proteins. The periplasmic domains of HrcC and HrcJ, i.e. the 34^th^ to the 370^th^ aa of HrcC and the 22^th^ to the 206^th^ aa of HrcJ, were therefore overexpressed and soluble fusion proteins were obtained, which were named His_6_-HrcC_34–370_ and His_6_-HrcJ_22–206_ (Fig. S5). His_6_-HpaM_LN22_ was biotinylated and immobilized on streptavidin sepharose beads. Pull-down assays between His_6_-HpaM_LN22_ and His_6_-HrcC_34–370_ or His_6_-HrcJ_22–206_ were performed (see methods for details). As shown in Fig. 4B, the protein HpaM_LN22_ did capture both His_6_-HrcC_34–370_ (lane 2) and His_6_-HrcJ_22–206_ (lane 4) proteins. Overall, these combined data demonstrate that HpaM interacts directly with the periplasmic domains of HrcC and HrcJ.

**Figure 5:**
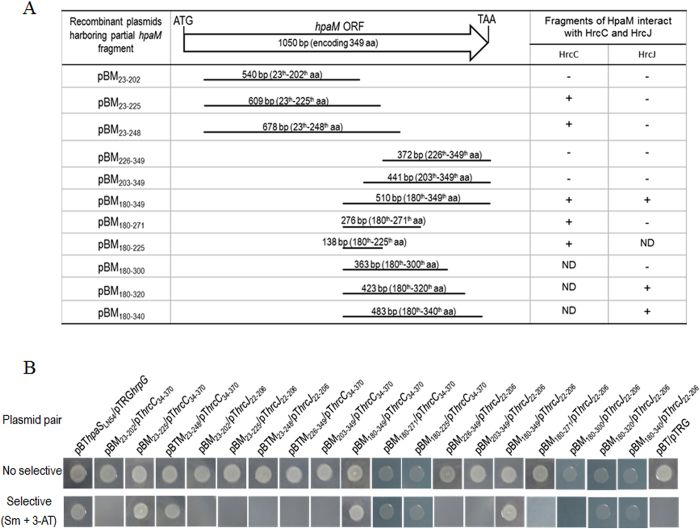
Determination of the peptides in HpaM required for the interaction with HrcC and HrcJ. (**A**) Schematic representation of a set of HpaM fragments used to test the interaction with HrcC or HrcJ. The left part of the figure shows the PCR fragments used to clone into the vector pBT and the resulting recombinant pBM series plasmids which were used for bacterial two-hybrid assays. The numbers above each line represent the length of PCR fragments and the corresponding region in HpaM. The right part of the figure shows the interaction between each of the truncated HpaM fragments and the periplasmic domain of HrcC or HrcJ. +, interaction; −, no interaction. ND, not done. (**B**) The results of bacterial two-hybrid assays. The plasmid pair pBT*hpaS*_LN54_/pTRG*hrpG* was used as a positive control.

To gain a primary insight into the molecular interaction between HpaM and HrcC or HrcJ, we defined the peptides in HpaM required for the interaction. As described above, the first 22 aa in the N-terminus of HpaM was predicted to be a signal peptide. We therefore tested the N-terminal portion exclusive of the first 22 aa. 540, 609, and 678 bp DNA fragments encoding the peptides of the 23^th^–202^th^ aa, 23^th^–225^th^ aa, and 23^th^–248^th^ aa, respectively, were amplified by using the corresponding primer sets listed in Table S2 and cloned into the vector pBT, respectively. 372, 441, 510, and 276 bp DNA fragments encoding the C-terminal peptides of the 226^th^–349^th^ aa, 203^th^–349^th^ aa, 180^th^–349^th^ aa, and 180^th^–271^th^ aa were also amplified and cloned into the vector pBT. The obtained recombinant plasmids (Table S1) as well as plasmid pT*hrcC*_34–370_ or pT*hrcJ*_22–206_ were introduced into the reporter strain XL1-Blue MRF′ and the growth of the resulting recombinant strains was examined. As shown in Fig. 5B, the recombinant strains that harbored the plasmid pair pBM_23–225_/pT*hrcC*_34–370_, pBM_23–248_/pT*hrcC*_34–370_, pBM_180–349_/pT*hrcC*_34–370_, pBM_180–271_/pT*hrcC*_34–370_ or pBM_180–349_/pT*hrcJ*_22–206_ could grow on the selective plate but other strains could not, indicating that the peptide consisting of the 180^th^ to the 225^th^ aa of HpaM is essential for the interaction between HpaM and HrcC, and the C-terminus of HpaM from the 180^th^ aa is involved in the interaction between HpaM and HrcJ. The 180^th^ to the 225^th^ aa of HpaM was further tested to see whether it suffices the interaction with HrcC, and the truncated proteins consisting of the 180^th^ aa to the 340^th^, the 320^th^, or the 300^th^ aa of HpaM were also further tested for their interactions with HrcJ, respectively. As shown in Fig. 5, the peptide from the 180^th^ to the 225^th^ aa of HpaM is sufficient for the interaction with HrcC (the strain containing pBM_180–225_/pT*hrcC*_34–370_ could grow on the selective agar plate), and the peptide from the 180^th^ to the 320^th^ (but not to the 300^th^) aa of HpaM is sufficient for the interaction with HrcJ (the strain containing pBM_180–320_/pT*hrcJ*_22–206_ could grow on the selective agar plate). The interactions were further confirmed by pull-down assays (Fig. 4B, lanes 12 and 14).

To evaluate whether the interaction with HrcC or HrcJ is essential for HpaM function, the HpaM derivatives with deletion in 180^th^–202^th^ aa consisting a PbH1 domain of parallel β-helix repeats and 288^th^–311^th^ aa consisting a PbH1 domain of parallel β-helix repeats, respectively, were constructed, and the obtained *hpaM* partial deletion mutants were named ΔhpaM_180–202_ and ΔhpaM_288–311_, respectively. Plant assays revealed that the two mutant strains, similar to the *hpaM* full deletion mutant ΔhpaM, scarcely caused any disease or HR symptoms in the host plant Chinese radish or the non-host plant pepper (Fig. S6). Additionally, the recombinant plasmid pLC*hpaM* carrying a full length *hpaM* gene was introduced into the mutants ΔhpaM_180–202_ and ΔhpaM_288–311_, respectively. The resulting complemented strains CΔhpaM_180–202_ and CΔhpaM_288–311_ showed wild-type virulence and HR phenotypes (Fig. S6).

### Evidences that HrcC, HpaM and HrcJ are outer and inner membrane-bound proteins, respectively, and HrcC of *Xcc* interacts directly with HrcJ

**Figure 6:**
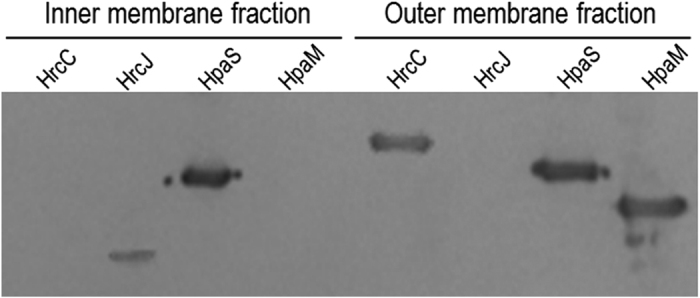
Evidence from western blot analysis reveals that HpaM, HrcC and HrcJ are outer and inner membrane-bound proteins, respectively. The outer and inner membrane fraction proteins from strain ∆hrcC/pR*hrcC*H6 (for HrcC detection), ∆hrcJ/pR*hrcJ*H6 (for HrcJ detection), and ΔHpaM-HrcC/pR*hpaM*H6 (for HpaM detection) were prepared. 10 μg of protein for each sample was separated by SDS-PAGE electrophoresis and transferred to a PVDF membrane. The presence of HrcC, HrcJ, and HpaM was detected by anti-His_6_ monoclonal antibody. The histidine sensor kinase HpaS (from strain ∆hpaS/pR*hpaS*H6) was used as a control.

In animal pathogens, the EscC/InvG/YscC family proteins compose of the outer membrane ring, and the EscJ/PrgK/YscJ family members are one of the inner membrane ring components. Periplasmic domains of EscC/InvG/YscC and EscJ/PrgK/YscJ proteins interact with each other and form the T3SS periplasmic rod^32^,^33^,^34^. HrcC and HrcJ in phytopathogens are isoforms of the EscC/InvG/YscC and EscJ/PrgK/YscJ families, respectively^6^. Deletion of *hrcC* or *hrcJ* abolished the virulence and HR induction of *Xcc* (Fig. S7). The N-termini of HrcC and HrcJ were predicted to be the periplasmic domains and their C-termini were supposed to integrate into the cell membranes. To verify the HrcC and HrcJ integration in *Xcc* cells, recombinant strains ∆hrcC/pR*hrcC*H6 and ∆hrcJ/pR*hrcJ*H6 were constructed, which produced HrcC and HrcJ with a 6×His tag on the C-terminus in the mutants ∆hrcC and ∆hrcJ, respectively. The outer and inner membrane protein fractions of the two strains grown to the late-log phase were prepared and exposed to western blot analysis. As shown in Fig. 6, HrcC and HrcJ were present in the outer and inner membrane fractions, respectively, indicating that HrcC and HrcJ in *Xcc*, as speculated, are outer and inner membrane-bounded proteins, respectively.

Our above data revealed that HpaM is an outer membrane-bound protein. As HrcC is believed to compose the outer membrane ring of the type III apparatus, we concerned that whether the outer membrane localization of HpaM depends on the presence of HrcC. We therefore detected the location of HpaM in the *hrcC* deletion mutant background. To do this, an *hpaM* and *hrcC* double deletion mutant named ∆hpaM-hrcC (Table S1) was constructed, and the recombinant plasmid pR*hpaM*H6 was introduced into the mutant. The resulting recombinant strain ∆hpaM-hrcC/pR*hpaM*H6 (Table S1) was used to locate HpaM protein. As shown in Fig. 6, HpaM protein was still present in the outer membrane fraction of the bacterial cells, indicating that the presence of HpaM in the outer membrane does not rely on HrcC, *i*.*e*. HpaM is in itself an outer membrane-bound protein.

To verify the *Xcc* HrcC and HrcJ proteins interact with each other, the truncated *hrcC* and *hrcJ* genes excluding the signal peptide coding sequence were cloned into the vector bait pBT and the prey pTRG, respectively, resulting the plasmids pB*hrcC*_34–370_ and pT*hrcJ*_22–206_ (Table S1). The plasmids were introduced into the reporter strain XL1-Blue MRF′. As shown in Fig. 4A, the strain harboring the plasmid pair pB*hrcC*_34–370_/pT*hrcJ*_22–206_ grew well on the selective indicator plate, while the strain harboring the plasmid pair pBT*hpaS*_LN54_/pT*hrcJ*_22–206_ or pB*hrcC*_34–370_/pTRG*hpaS*_LN54_ could not grow. These results indicate that the interaction between HrcC and HrcJ existed. Protein pull-down assay was carried out to further verify the bacterial two-hybrid assay result. His_6_-HrcC_34–370_ and His_6_-HrcJ_22–206_ were biotinylated with sulfo-NHS-LC-biotin and incubated with streptavidin sepharose™ beads, respectively, and then the protein His_6_-HrcJ_22–206_ or His_6_-HrcC_34–370_ was added. As shown in Fig. 4B, His_6_-HrcC_34–370_ and His_6_-HrcJ_22–206_ captured each other (lane 6 and 10). Additionally, both proteins His_6_-HrcC_34–370_ and His_6_-HrcJ_22–206_ were able to capture the protein His_6_-HpaM_LN22_ (Fig. 4B, lane 7 and 11). These combined data confirm that HrcC and HpaM are outer membrane-bound proteins, HrcJ is an inner membrane-bound protein, and HrcC and HrcJ interact with each other directly in *Xcc*.

### HpaM is highly conserved in phytopathogenic Xanthomonads

To date, the whole genome sequences of more than one dozen *Xanthomonas* spp. or pathovars are available. A protein blast revealed that HpaM is conserved in all sequenced *Xanthomonas* spp. (Table S3). Although the rate of their amino acid sequence homology is varied among different species or pathovars, most of which share more than 90% similarity and 87% identity. Only three species, i.e., *X. translucens, X. sacchari*, and *X. albilineans*, share an HpaM homologue with lower similarity (71–74%) and identity (about 60%) to *Xcc* HpaM. Additionally, an HpaM homologue also exists in *Pseudoxanthomonas spadix* and *Xylella fastidiosa*, which shares ~55% identity and ~68% similarity with *Xcc* HpaM (Table S3). Transmembrane domain analysis using the TMPRED program (http://www.ch.embnet.org/software/TMPRED_form.html) revealed that the N-termini of all the HpaM homologues contain a transmembrane helice (Table S3).

### The *Xanthomonas oryzae* homologues of HpaM exhibit similar functions to *Xcc* HpaM

As described above, HpaM is highly conserved in *Xanthomonas* pathogens. To verify whether the HpaM homologues in other *Xanthomonas* spp. play similar roles to *Xcc* HpaM, we investigated the function of the HpaM homologues in the species *Xanthomonas oryzae. X. oryzae* consists of two pathovars, *oryzae (Xoo*) and *oryzicola (Xoc*), which are the causative agents for bacterial leaf blight and bacterial leaf streak of rice, respectively. The whole-genome sequences are available for *Xoo* strain PXO99^A ^35 and *Xoc* strain GX01 (our unpublished data), therefore, we used these strains in the study. The HpaM homologues in strain PXO99^A^ and strain GX01 were designated as HpaM_Xoo_ and HpaM_Xoc_, respectively. HpaM_Xoc_ is completely identical to its counterpart in the *Xoc* strain BLS256^36^. If HpaM_Xoo_ and HpaM_Xoc_ are entrusted with similar functions to *Xcc* HpaM, they should be able to replace *Xcc* HpaM and restore the virulence and HR induction of the *Xcc hpaM* deletion mutant. Therefore, we cloned the *hpaM* homologues of *Xoo* and *Xoc* into the vector pLAFR3 (Table S1) and introduced the resulting recombinant plasmids pLC*hpaM*_Xoo_ and pLC*hpaM*_Xoc_ (Table S1) into the *Xcc hpaM* deletion mutant strain ΔhpaM, respectively. Plant tests showed that either of pLC*hpaM*_Xoo_ and pLC*hpaM*_Xoc_ could restore the ability of the mutant to induce typical black rot symptoms in the host plant Chinese radish and HR in the non-host plant pepper leaves (Fig. 1A,B, Fig. S8), indicating that *Xcc* HpaM and its counterparts in *Xoo* and *Xoc* probably have similar functions.

**Figure 7:**
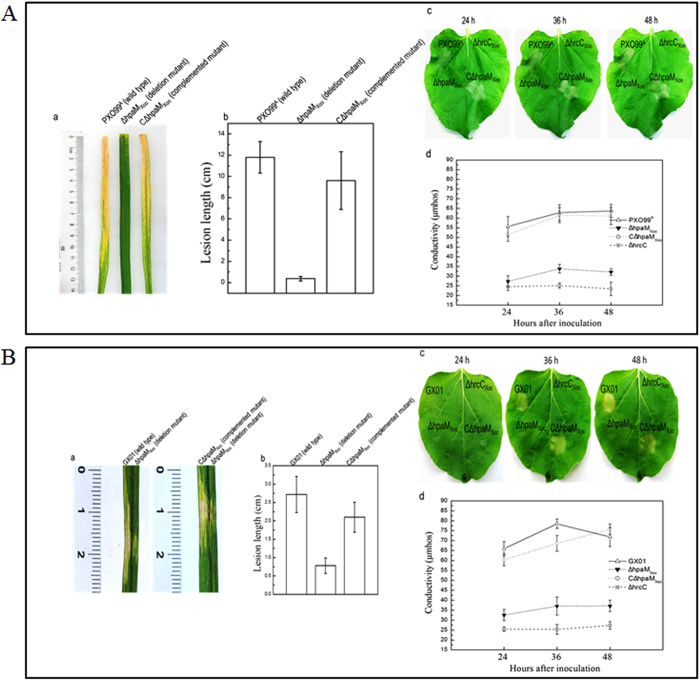
HpaM homologues in *Xoo* and *Xoc* are critical for virulence and HR induction. Bacterial cells were cultured in NB medium and resuspended in sterile distilled water to a concentration of OD_600_ of 0.3 (for virulence assay) or 0.5 (for HR assay). For virulence test, the bacterial resuspensions were inoculated onto 6-week-old leaves of rice plant (*Oryza sativa* L. ssp. *Japonica* cultivar *Nipponbare*) by the leaf-clipping method (for *Xoo*) or by infiltrating with needleless syringe (for *Xoc*). For HR induction, the bacterial resuspensions were infiltrated into tobacco (*Nicotina benthamiana*) leaf mesophyll tissue. (**A**) *Xoo* strains; (**B**) *Xoc* strains. (**a**) Disease symptoms 14 days after inoculation; (**b**) lesion lengths scored 14 days after inoculation. Values represent means and standard deviation from twenty inoculated leaves in one experiment. The experiment was repeated three times with similar results. (**c**) HR symptoms photographed at 24, 36 and 48 h after infiltration. The *hrcC* deletion mutant strains ΔhrcC_Xoo_ (derivative of *Xoo*) and ΔhrcC_Xoc_ (derivative of *Xoc*) were used as negative controls. Three replications were done in each experiment and the experiment was repeated three times. The results presented are from a representative experiment, and similar results were obtained in all other independent experiments. (**d**) Electrolyte leakage from tobacco leaves inoculated with *Xoo* or *Xoc* strains. For each sample, four 0.4 cm^2^ leaf disks were collected from the bacteria-inoculated area and incubated in 5 ml distilled water. Conductivity was measured with a DDS-307A conductometer. Three samples were taken for each measurement in each experiment. Results presented are from a representative experiment, and similar results were obtained in two other independent experiments.

**Figure 8:**
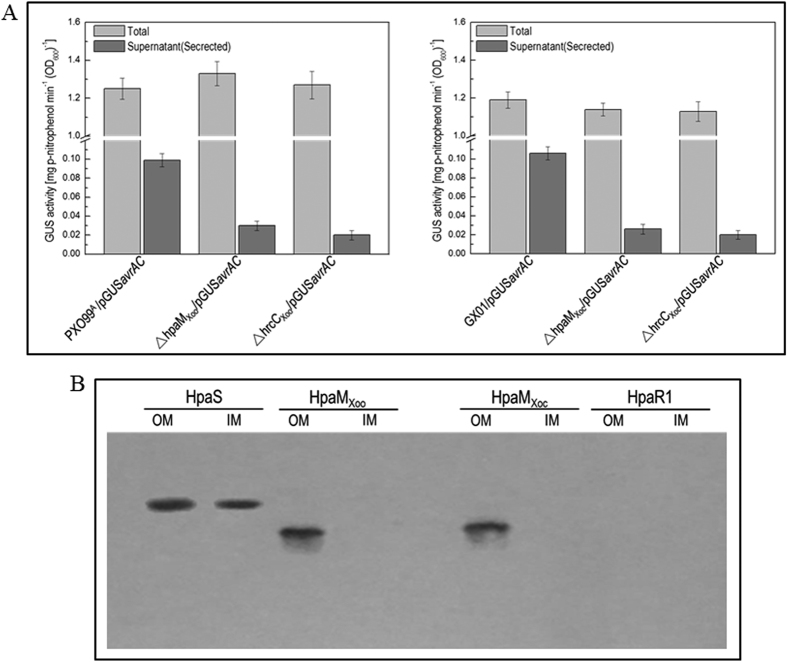
HpaM homologues in *Xoo* and *Xoc* have similar functions to HpaM. (**A**) HpaM_Xoo_ (HpaM homologue in *Xoo*) and HpaM_Xoc_ (HpaM homologue in *Xoc*) are essential for type III secretion. Type III secretion signal sequence-*gusA* fusion reporter plasmid pGUS*avrAC* was introduced into *Xoo* and *Xoc* strains. The resulting recombinant strains were cultured in XOM2 medium for 12 h and the β-glucuronidase (GUS) activities in the culture (Total) and the cultural supernatant (Secreted) were determined. Values are the means ± standard deviation from three repeats. Left and right elements, GUS activities produced by pGUS*avrAC* in *Xoo* and *Xoc* strains, respectively. (**B**) The HpaM homologues HpaM_Xoo_ and HpaM_Xoc_ are also located in the outer membrane. The outer and inner membrane fraction proteins from *Xoo* and *Xoc* strains were prepared and 10 μg of each protein sample was separated by SDS-PAGE electrophoresis and transferred to a PVDF membrane. The presence of tested proteins was detected by anti-His_6_ monoclonal antibody. The histidine sensor kinase HpaS and transcriptional regulator HpaR1 of *Xcc* were used as positive and negative controls. OM, outer membrane; IM, inner membrane.

To further investigate the function of HpaM_Xoo_ and HpaM_Xoc_ in *Xoo* and *Xoc, hpaM*_*Xoo*_ and *hpaM*_*Xoc*_ deletion mutants were constructed from strain PXO99^A^ and strain GX01 by homologous recombination using the suicide vector pK18mob*sacB*^37^, and the resulting mutants, named ΔhpaM_Xoo_ and ΔhpaM_Xoc_ (Table S1), were tested for virulence in rice and HR in tobacco. As shown in Fig. 7, both mutants almost completely failed to stimulate disease symptoms in rice and HR in tobacco, while the complemented strains could induce wild-type disease symptoms and HR. As the T3SS is also essential for the pathogenicity and HR induction of both pathogens, the plant test result suggests that HpaM is probably indispensable for a functional T3SS of *Xoo* and *Xoc*. To verify this, the type III secretion efficiency of the mutants ΔhpaM_Xoo_ and ΔhpaM_Xoc_ was detected. To do this, the type III secretion reporter plasmid pLGUS*avrAC* (Table S1) was introduced into the mutants ΔhpaM_Xoo_ and ΔhpaM_Xoc_ as well as the wild type strains of *Xoo* and *Xoc*. The GUS activities of the resulting recombinant strains were then determined. As shown in Fig. 8A, both mutants harboring pLGUS*avrAC* produced significantly weaker GUS activity in cultural supernatants, compared to the wild type strains harboring pLGUS*avrAC*, suggesting that the type III secretion efficiency of the mutants was significantly weakened. These combined data demonstrate that the HpaM homologues of *Xoo* and *Xoc* are also critical for the type III secretion. The cellular location of HpaM_Xoo_ and HpaM_Xoc_ was also determined by western blot assay. The HpaM_Xoo_ and HpaM_Xoc_ encoding sequences fused with 6×His tag at their C-termini were cloned into pLAFR3 and the resulting recombinant plasmids named pR*hpaM*_*Xoo*_H6 and pR*hpaM*_*Xoc*_H6 (Table S1) were introduced into the mutant strains ΔhpaM_Xoo_ and ΔhpaM_Xoc_, respectively. The outer and inner membrane proteins from the obtained recombinant strains ΔhpaM_Xoo_/pR*hpaM*_*Xoo*_H6 and ΔhpaM_Xoc_/pR*hpaM*_*Xoc*_H6 (Table S1) were prepared and analyzed by western blot assay. The result revealed that HpaM_Xoo_ and HpaM_Xoc_ were also located in the outer membrane of *Xoo* and *Xoc* (Fig. 8B). Taken together, the above combined data indicate that the *Xoo* and *Xoc* homologues of HpaM may have similar functions to *Xcc* HpaM.

## Discussion

Here we have demonstrated that the novel outer membrane-bound protein HpaM is critical for the type III secretion of *Xanthomonas* spp. Mutation of *hpaM* did not alter the production of extracellular enzymes and polysaccharides as well as cell motility, suggesting that HpaM may specifically affect the T3SS. HpaM is not involved in the regulation of the expression of *hrp* genes that encode the components of the T3SS machinery, but interacts with HrcC and HrcJ, the homologues of the components that compose the outer and inner rings of the T3SS basal body of all bacterial pathogens that possess a T3SS. Mutation of *hrcC* or *hrcJ* almost completely broke the type III secretion of *Xcc*, resulting in loss of the ability to cause disease symptoms and HR. In animal pathogens, it has been shown that the outer and inner ring proteins are outer and inner membrane proteins, respectively, and they physically interact with each other directly^5^,^6^,^7^,^8^. In this work, we authenticated that *Xcc* HrcC and HrcJ, as expected, are located in the outer and inner membrane, respectively, and they interact with each other directly. These data provide supporting evidence to the inference that HrcC and HrcJ act as T3SS outer and inner ring proteins in *Xanthomonas* spp. The peptide consisting of 46 amino acids from the 180^th^ to 225^th^ aa of HpaM is sufficient for interaction with HrcC but not HrcJ, and the most portion of the C-terminus, containing the amino acids from the 180^th^ to 320^th^ aa, is indispensable for the interaction with HrcJ. Bioinformatics analysis revealed that transmembrane helices are present in the N-terminus of HpaM. Taken together, these data suggest that HpaM is integrated into the outer membrane with its N-terminal domain and extends into the periplasm, where its middle part interacts with the outer ring protein HrcC and the C-terminal portion including the middle part interacts with the inner ring protein HrcJ, forming a three protein complex. It is worth noting that HpaM is predicted to have six right-handed parallel β-helix repeats from the 120^th^ to 311^th^ residues (i.e. 120–163, 180–202, 203–225, 226–248, 249–271, and 288–311), five of which lie in the region related to its physical interaction with HrcC and HrcJ. The right-handed parallel β-helix repeats are most commonly associated with autotransporter proteins, many of which are extracellular enzymes. It is clear that the β-helix repeats are essential not only for protein folding but also for functions, such as forming an appropriate structure that recognizes the substrates^38^,^39^. The presence of the β-helix repeats within the region interacting with HrcC and HrcJ suggests that they may be critical for HpaM stability and the formation of the protein complex.

Comparative bioinformatics analysis revealed that HpaM is conserved in all sequenced *Xanthomonas* species. To expand our knowledge on the function of HpaM in other *Xanthomonas* spp., we also investigated the HpaM homologues in *X. oryzae* pathovars *oryzae* and *oryzicola*. The results demonstrated that the HpaM homologues in the two pathovars of *X. oryzae* also localize in the outer membrane and are critical for pathogenicity and HR as well as efficient type III secretion. Furthermore, they can replace HpaM in *Xcc* for the type III secretion. These results indicate that HpaM is conserved not only in structure but also in function in *Xanthomonas* spp. Interestingly, HpaM homologs are also present in the species *Pseudoxanthomonas spadix* and *Xylella fastidiosa* (Table S3). Like the genus *Xanthomonas, Pseudoxanthomonas* and *Xylella* genera also belong to the family *Xanthomonadaceae*. It is possible that HpaM homologues are also prevalent in the members of these genera. However, *Pseudoxanthomonas spadix* and *Xylella fastidiosa* do not seem to have a T3SS. To investigate the function of the HpaM homologues in these bacteria will be a valuable topic.

At this stage, the precise role of HpaM in the T3SS is not clear. However, given the facts that: 1) HpaM does not act as a regulator for *hrp* gene expression but is critical for type III secretion; 2) its N-terminus integrates in the outer membrane and C-terminus extends deeply into the periplasm to interact physically with the inner ring protein HrcJ; and 3) its middle part interacts physically with the outer ring protein HrcC, forming a HpaM-HrcC-HrcJ complex, we presume that HpaM is most likely to be a structural component of the T3SS in *Xanthomonas* spp., although it is not encoded by a gene within the *hrp* cluster. In general, T3SS structural components of animal and plant pathogens are encoded by chromosomal or plasmid-borne gene clusters that were probably acquired during evolution by horizontal gene transfer^6^,^40^. As described above, the cluster consists of more than 20 genes and nine of which are conserved among plant and animal pathogens. These conserved genes are believed to encode the core components of the T3SS machinery in both plant and animal pathogens. However, more than 50% of the genes in the clusters are varied, suggesting that the clusters have changed a lot in different pathogens during the long-term evolution. A phylogenetic tree analysis divides the T3SSs of plant and animal pathogens into at least six families including two families from plant pathogens^41^. Therefore, it is possible that although the architectures of the T3SS apparatuses in different pathogens are similar, some fittings may not be the same. As described above, the T3SS extracellular appendage (pilus-like) of plant pathogens is different from that (needle-like) of animal pathogens. In addition, the inner rod may be another case showing different fittings in the T3SSs between plant and animal pathogens. The inner rod is a part of the T3SS basal body found in animal pathogens, which is formed by a periplasmic protein that connects the outer and inner rings^33^,^34^. However, the inner rod homologous protein is missing in plant pathogens. A non-homologous protein, HrpB2, has been supposed to be a putative inner rod protein of *X. euvesicatoria*, based on the features that it contains a VxTLxK amino acid motif that is conserved in the inner rod proteins of animal pathogens, localizes to the periplasm and the outer membrane, and is essential for T3SS pilus formation^6^,^42^.

A periplasmic protein, named VrpA, encoded by a gene outside the *hrp* cluster of *X. citri* subsp. *citri*, was recently reported to contribute to the secretion efficiency of the T3SS^43^. Similar to HpaM, VrpA is conserved in *Xanthomonas* spp. and also physically interacts with HrcC and HrcJ but not HrpB2. It was presumed that VrpA may affect activation of secretion and assembly or stability of the T3SS apparatus via interacting with HrcC and HrcJ^43^. We cannot exclude the possibility that HpaM associates with the T3SS via assisting the apparatus assembling or affecting the apparatus stability rather than as a T3SS structural component. Nonetheless, given the fact that HpaM as well as VrpA are *Xanthomonas* genus-specific proteins which are absent in other bacterial pathogens that possess a T3SS, our results suggest that the T3SS of *Xanthomonas* is distinctive in some aspects from other pathogens. To further investigate the precise role of HpaM and VrpA will greatly facilitate our understanding of the T3SS biogenesis.

## Materials and Methods

### Bacterial strains, plasmids and growth conditions

The bacterial strains and plasmids used in this study are listed in Table S1. *Escherichia coli* strains were grown in Luria–Bertani medium^44^ at 37 °C. *Xcc* strains were grown at 28 °C in NYG medium^45^, the minimal medium MMX^46^ or XVM2^31^. *Xoo* and *Xoc* strains were grown at 28 °C in OB medium^47^, NB medium^48^, or the minimal medium XOM2^49^. Antibiotics were added at the following concentrations as required: kanamycin (Kan) 25 μg ml^−1^, rifampicin (Rif) 50 μg ml^−1^, ampicillin (Amp) 100 μg ml^−1^, spectinomycin (Spc) 50 μg ml^−1^, gentamicin (Gm) 5 μg ml^−1^, streptomycin (Sm) at 100 μg ml^−1^, and tetracycline (Tet) 5 μg ml^−1^ for *Xanthomonas* spp. and 15 μg ml^−1^ for *E. coli*.

### DNA and RNA techniques, SDS-PAGE and western blotting

DNA manipulations followed the procedures described by Sambrook and associates^50^. Plasmids were transformed into cells of *E. coli* and *Xanthomonas* spp. by electroporation or conjugation described by Turner and associates^51^. The restriction endonucleases, T4 DNA ligase, and *pfu* polymerase were provided by Promega (Shanghai, China). The total RNAs from *Xanthomonas* spp. were extracted with a total-RNA extraction kit (Promega), and reverse transcription was performed using a cDNA synthesis kit (Fermentas Co., Vilnius, Lithuania). Each kit was used according to the manufacturer’s instructions.

Western blotting was carried out as previously described^21^. Briefly, bacterial proteins were separated by 12% (w/v) SDS-PAGE and transferred onto PVDF (polyvinylidene difluoride) membrane (Millipore Corporation, Billerica, MA, USA). After blocking, the 1:2500 diluted anti-His-tag mouse monoclonal antibody (Qiagen, Shanghai, China) was used as the primary antibody, and the 1:2500 diluted horseradish peroxidase conjugated goat antimouse IgG (Bio-Rad, Hercules, CA, USA) was used as secondary antibody.

### Deletion mutant construction and complementation

The *hpaM* in *Xcc* and its homologues *hpaM*_Xoo_ (in *Xoo*) and *hpaM*_Xoc_ (in *Xoc*) were deleted by the method described by Schäfer and associates^37^. For construction of *Xcc hpaM* deletion mutant, 747-bp upstream and 726-bp downstream fragments flanking *hpaM (XC*_*2847*) were amplified with the primer sets L*hpaM*-F/R and R*hpaM*-F/R (Table S2), respectively, using the total DNA of the *Xcc* wild type strain 8004 as a template. Primers were modified to give *Eco*RI-, *Xba*I- or *Hin*dIII-compatible ends (underlined) (Table S2). The two fragments were cloned together into the vector pK18*mobsacB*^37^, and the resulting plasmid named pK18mobsacBhpaM was introduced into the *Xcc* strain 8004 by triparental conjugation. The transconjugants were screened on selective agar plates containing 5% sucrose. The obtained *hpaM* deletion mutant was further confirmed by PCR and named ΔhpaM.

The HpaM derivatives with deletion in 180^th^–202^th^ aa or 288^th^–311^th^ aa were constructed by using the same method. For the HpaM derivative with deletion in 180^th^–202^th^ aa, a 767-bp fragment spanning the 230^th^ nt upstream to the 537^th^ nt downstream of the start codon ATG of *hpaM* ORF and a 560-bp fragment spanning the 607^th^ nt to the 1166^th^ nt downstream of the start codon ATG of *hpaM* ORF were amplified with the primer sets L*hpaM*-F_180_/R_180_ and R*hpaM*-F_180_/R_180_. The resulting *hpaM* partial deletion mutant was named ΔhpaM_180–202_. For the HpaM derivative with deletion in 288^th^–311^th^ aa, a 715-bp DNA fragment spanning the 147^th^ nt to the 861^th^ nt downstream of the start codon ATG of *hpaM* ORF and a 588-bp DNA fragment spanning the 934^th^ nt to the 1521^th^ nt downstream of the start codon ATG of *hpaM* ORF were amplified with the primer sets L*hpaM*-F_311_/R_311_ and R*hpaM*-F_311_/R_311_. The resulting *hpaM* partial deletion mutant was named ΔhpaM_288–311_.

For deletion of *hpaM*_Xoo_ (*PXO*_*01147*) or *hpaM*_Xoc_ (*XOC*_*3053* homologue), 879-bp upstream and 591-bp downstream fragments flanking *hpaM*_Xoo_ or *hpaM*_Xoc_ were amplified with the corresponding primer sets (Table S2) from the *Xoo* strain PXO99^A^ and the *Xoc* strain GX01, respectively. The resulting deletion mutants were named ΔhpaM_Xoo_ and ΔhpaM_Xoc_ (Table S1). For complementation of the *hpaM* deletion mutant, a 1432-bp DNA fragment containing the *hpaM* coding region and extending from 352-bp upstream of the 5′ end to 30-bp downstream of the 3′ end of the ORF was amplified by PCR from the total DNA of the *Xcc* strain 8004 with the primer set C*hpaM*-F/R (Table S2). Primers were modified to give *Bam*HI- or *Hin*dIII-compatible ends (underlined) (Table S2). The amplified fragment was confirmed by sequencing, and ligated into the promoterless cloning site of the plasmid pLAFR6^52^, generating the recombinant plasmid named pLC*hpaM* (Table S1). The plasmid was introduced into the *hpaM* deletion mutant or partial deletion mutants by triparental conjugation, generating complemented strains named CΔhpaM, CΔhpaM_180–202_ and CΔhpaM_288–311_, respectively (Table S1). 1053-bp DNA fragments of the *hpaM*_Xoo_ (*PXO*_*01147*) ORF and *hpaM*_Xoc_ (*XOC*_*3053* homologue) ORF were also amplified by PCR from the *Xoo* strain PXO99^A^ and the *Xoc* strain GX01, respectively, and cloned into the plasmid pLAFR3^53^. The resulting recombinant plasmids named pLC*hpaM*_Xoo_ and pLC*hpaM*_Xoc_ (Table S1) were used to complement the mutant strains ΔhpaM, ΔhpaM_Xoo_, and ΔhpaM_Xoc_.

For construction of *avrAC (XC*_*1553*) deletion mutant, *avrAC-hpaM* double deletion mutant and *avrAC-hrcV* double deletion mutant, 577-bp upstream and 461-bp downstream fragments flanking the ORF *XC*_*1553 (avrAC*) were amplified with the primer sets L*avrAC*-F/R and R*avrAC*-F/R (Table S2). The two fragments were cloned together into the *Bam*HI/*Hin*dIII sites of pK18*mobsacB*^37^. The resulting recombinant plasmid pK18mob*sacBavrAC* was introduced into the *Xcc* wild type strain 8004, the *hpaM* mutant strain ΔhpaM, and the *hrcV* mutant strain ΔhrcV, respectively. The obtained mutants were named ΔavrAC, ΔavrAC-hpaM, and ΔavrAC-hrcV, respectively. To complement these mutants, a 2196-bp DNA fragment of *avrAC* gene (including 588 bp upstream sequence and *avrAC* coding sequence) fused with 6×His tag encoding sequences was amplified with the primer set H*avrAC*-F/R (Table S2). The obtained PCR fragment was cloned into the promoterless *Bam*H/*Pst*I sites of pLAFR6. The resulting recombinant plasmid pR*avrAC*H6 was introduced into the mutant strain ΔavrAC, ΔavrAC-hpaM, and ΔavrAC-hrcV, respectively, generating recombinant strains ∆avrAC/pR*avrAC*H6, ∆avrAC-hpaM/pR*avrAC*H6, and ∆avrAC-hrcV/pR*avrAC*H6 (Table S1).

### Determination of transcriptional start site

To determine the transcriptional start site of the *hpaM* gene, 5′-RACE (5′ rapid amplification of cDNA ends) method was carried out with the *hpaM* sequence-specific primers *hpaM*-RTP1-4 (Table S2). The assay was performed as previously described^21^. Briefly, total cellular RNA was extracted from the *Xcc* wild type strain 8004 grown in NYG medium to an OD_600_ of 1.0. cDNA fragments were obtained using the 5′-RACE kit (Invitrogen Life Technologies, San Diego, CA, USA), and PCR products were cloned into the vector pMD19-T and sequenced.

### Construction of promoter reporter plasmid

A promoter reporter plasmid for *hpaM* was constructed by fusing a 404-bp DNA fragment upstream of *hpaM* ORF (including the translation start codon ATG) with the promoterless β-glucuronidase (GUS)-encoding ORF (excluding the translation start codon ATG). The *hpaM* promoter region was amplified from the total DNA of the *Xcc* wild type strain 8004 by using the primer set RP-*hpaM*F/R (Table S2). The *gusA* coding region was amplified by PCR with the primer set GusF/R (Table S2), using the transposon Tn5*gusA*5 DNA as template. Primers were modified to give *Eco*RI-, *Bam*HI- or *Pst*I- compatible ends (underlined) (Table S2). The two fragments obtained were cloned into the promoterless cloning sites of the plasmid pLAFR6 to generate the reporter plasmid named pGUS*hpaM* (Table S1).

### Bacterial two-hybrid assay

The BacterioMatch II two-hybrid system (Stratagene, La Jolla, CA, USA) was used to detect protein-protein interaction *in vivo*. The truncated (or full length) *hpaM, hrcC* and *hrcJ* were amplified by PCR using the total DNA of the *Xcc* wild type strain 8004 as template and corresponding oligonucleotide set as primers (Table S2), respectively. The 981 bp truncated *hpaM* gene (from the 67^th^ to the 1047^th^ nt of the *hpaM* gene coding sequence, excluding the signal peptide coding sequence) was cloned into the *Bam*HI/*Xho*I sites of pBT (bait), generating the plasmid pB*hpaM*_LN22_ (Table S1). 1716- and 1011-bp fragments of truncated *hrcC*, and 699- and 555-bp fragments of truncated *hrcJ* were cloned into the *Bam*HI/*Xho*I sites of the vector pTRG (prey), respectively, generating the plasmids named pT*hrcC*_LN33_, pT*hrcC*_34–370_, pT*hrcJ*_LN21_ and pT*hrcJ*_22–206_ (Table S1). The bacterial two-hybrid assay was performed according to the manufacturer’s instructions. To test the interaction of a variety length of HpaM fragments with the periplasmic domain of HrcC and HrcJ, 540, 609, 678, 372, 441, 510, 276, 138, 363, 423 and 483-bp DNA fragments containing partial *hpaM* gene were amplified by PCR using the corresponding primer sets (Table S2), respectively, and the obtained DNA fragments were cloned into *Bam*HI/*Xho*I sites of pBT, resulting a series of pBM recombinant plasmids (Table S2). The plasmid pairs (Fig. 5) were used to co-transform the reporter strain XL1-Blue MRF′ on M9 salt agar without 3-AT. Colonies were then restreaked on M9 salt agar containing 5 mM 3-AT and incubated at 37 °C for 24 h for the first detection of interaction. For confirmation, the colonies were cultured on dual selective medium containing 5 mM 3-AT and 12.5 μg ml^−1^ Sm, as described in the manual.

### Overproduction and purification of proteins

To overproduce 6×His-tagged truncated forms of HpaM, 981, 138 and 423-bp DNA fragments encoding 23^th^–349^th^ (excluding signal peptide), 180^th^–225^th^ and 180^th^–320^th^ amino acids were amplified by using the primer set *hpaM*-OF/R, *hpaM*O-9F/R and *hpaM*O-11F/R (Table S2), respectively. The obtained DNA fragments were cloned into *Bam*HI/*Hin*dIII sites of the expression vector pET-30a (Novagen), resulting the recombinant plasmid named pET-30a-HpaM_LN22_, pET-30a-HpaM_LN180–225_ and pET-30a-HpaM_LN180–320_, respectively (Table S1). The recombinant plasmids were then transformed into *E. coli* strain BL21 (DE3), resulting strains BL21/pET-30a-HpaM_LN22_, BL21/pET-30a-HpaM_LN180–225_ and BL21/pET-30a-HpaM_LN180–320_. After cultivation and induction by IPTG (isopropyl-thiogalactopyranoside), the cells were harvested and 6×His-tagged fused proteins were purified by Nickel-NTA resin (Qiagen). For overproduction of the periplasmic domain of HrcC and HrcJ, a 1011 bp DNA fragment encoding the 34^th^–370^th^ amino acids of HrcC and a 555-bp fragment encoding the 22^th^–206^th^ amino acids of HrcJ were amplified by using the primer sets *hrcC*-N2F/R and *hrcJ*-N2F/R (Table S2), respectively. The resulting fragments were cloned into the *Bam*HI/*Hin*dIII sites of pET-30a (Novagen), generating the recombinant plasmids named pET-30a-HrcC_34–370_ and pET-30a-HrcJ_22–206_ (Table S1). The recombinant plasmids were transformed into *E. coli* strain BL21 (DE3), resulting recombinant strains BL21/pET-30a-HrcC_34–370_ and BL21/pET-30a-HrcJ_22–206_, respectively (Table S1).

### Protein pull-down assay

Protein pull-down assay was performed as previously described^21^, with the ProFound pull-down biotinylated protein-protein interaction kit (Pierce, Rockford, IL, USA). The *hpaM* fusion protein His_6_-HpaM_LN22_ was biotinylated with sulfo-NHS-LC-biotin, and the labeled protein was purified by dialysis. 50 μl of the purified biotinylated His_6_-HpaM_LN22_ (0.5 mg ml^−1^) was incubated with 40 μl of streptavidin sepharose™ beads. After centrifugation, beads were washed three times with binding buffer containing 300 mM NaCl and 100 μl of sample containing 50 μg suspected prey protein (His_6_-HrcC_34–370_ or His_6_-HrcJ_22–206_) was added. After incubation at 4 °C for at least 60 min, beads were washed with wash buffer and prey protein was eluted in 150 μl elution buffer. 20 μl of the eluted sample was eletrophored on 12% SDS-PAGE gel and visualized by coomassie blue staining. For detection of HrcC-HrcJ interaction, His_6_-HrcC_34–370_ and His_6_-HrcJ_22–206_ were overproduced, purified and biotinylated with sulfo-NHS-LC-biotin, followed by incubation with streptavidin sepharose™ beads, respectively. His_6_-HrcC_34–370_ and His_6_-HrcJ_22–206_ proteins were then added into the above biotinylated His_6_-HrcJ_22–206_ and His_6_-HrcC_34–370_, respectively. For detection of the interaction between His_6_-HrcC_34–370_ (or His_6_-HrcJ_22–206_) and each of the truncated HpaM fragments, His_6_-HpaM_LN22_, His_6_-HpaM_LN180–225_, or His_6_-HpaM_LN180–320_ was added.

### GUS activity assay

GUS activity was determined by measurement of the absorbance of OD_415_ using ρ-nitrophenyl-β-D-glucuronide as the substrate, as described by Henderson and associates^54^, after growth of bacterial cells in medium for a period of time. To determine the GUS activity of secreted proteins, the bacterial cells of 200 μl culture for each strain were separated by centrifugation and the cell-free supernatant was taken for GUS activity determination.

### Plant assay

The virulence of *Xcc* to Chinese radish (*Raphanus sativus*) was tested by the leaf-clipping method^55^. Bacterial cells from overnight culture were collected, washed with 10 mM sodium phosphate buffer (SPB, 5.8 mM Na_2_HPO_4_ and 4.2 mM NaH_2_PO_4_, pH 7.0) and resuspended in the same buffer to an OD_600_ of 0.1 (1 × 10^8^ CFU ml^−1^). Leaves were cut with scissors dipped in the bacterial suspensions. Lesion length was measured 10 days after inoculation, and data were analysed by *t*-test. The HR was tested on the pepper plant ECW-10R (*Capsicumannuum* cv. ECW-10R) as previously described^21^. For each *Xcc* strain tested, an approximately 5 μl bacterial resuspension (1 × 10^8^ CFU ml^−1^) was infiltrated into the abaxial leaf surface of pepper plant. The inoculated plants were maintained in appropriated conditions, and HR symptoms were observed and photographed at 8, 16 and 24 h after inoculation. For the electrolyte leakage assay, bacterial cells were resuspended in sterile distilled water at a concentration of OD_600_ of 0.1. Four 0.4 cm^2^ leaf disks for each sample were collected from the bacteria-infiltrated area and incubated in 5 ml of distilled water. Conductivity was measured with a DDS-307A conductometer.

For *Xoo* virulence assay, the wild-type strain PXO99^A ^56 and its derivatives were tested on susceptible rice plant *Oryza sativa* L. ssp. *Japonica* cultivar *Nipponbare* using leaf clip inoculation method^57^. For *Xoc*, the wild type strain GX01 and its derivatives were infiltrated into rice leaves by needleless syringe^58^. Bacterial cells were grown for 72 h at 28 °C in NB medium with appropriate antibiotics. The cells were collected and resuspended in sterile distilled water to a concentration of OD_600_ = 0.3. Inoculation was carried out on 6-week-old rice plants under relevant conditions. Symptoms were recorded by photography and the disease lesion lengths were measured 14 days after inoculation. Twenty-five leaves were inoculated for each strain in each experiment. The experiment was repeated three times.

The HR test of *Xoo* and *Xoc* was conducted as described by Guo *et al*.^59^ and Zou *et al*.^60^, respectively. Briefly, *Xoc* or *Xoo* strains were cultured in NB medium to logarithmic phase, and cells were pelleted and suspended in water to a concentration of OD_600_ = 0.5. The suspensions were infiltrated into leaves of glass house-grown tobacco (*Nicotina benthamiana*), and the results were observed at 24, 36 and 48 h after infiltration. If the strain had the ability to trigger HR, the phenomenon of programmed cell death would be observed around the inoculation sites on tobacco leaves. The detection of the electrolyte leakage in tobacco leaves inoculated with *Xoo* or *Xoc* strains was similar to the method used to detect that in pepper leaves inoculated with *Xcc* strains.

### Cya protein translocation assay

To determine Cya enzyme activity *in vivo*, a modification of the procedure described by Roden and associates^61^ was carried out. Briefly, a 894-bp DNA fragment spanning nucleotides 588-bp upstream to 306-bp downstream of the translation start codon ATG of *avrAC (XC*_*1553*) was fused with the ORF of *cyaA* (excluding the translational start codon ATG) and ligated into pLAFR6, resulting the plasmid pL*avrAC*_102_::CyaA (Table S1). The plasmid was then introduced into *Xcc* wild type, *hpaM* mutant, and *hrcV* mutant strains. The resulting recombinant strains were cultured in NYG medium, and bacterial cells from the cultures were resuspended in 10 mM MgCl_2_ to a concentration of OD_600_ of 0.1 and then infiltrated into 20 plant leaves. A direct cyclic AMP (cAMP) correlation enzyme immunoassay kit (Amersham) was used to process the leaf samples and measure the cAMP concentrations following the manufacturer’s instructions. The protein content of each sample was determined using the Bio-Rad protein assay (Bio-Rad). Cya enzyme activity was expressed as pmol of cAMP per mg of total protein.

### Preparation of total, periplasmic, and outer membrane proteins

The bacterial total and periplasmic proteins were prepared using the method described previously^62^. The bacterial outer and inner membrane proteins were prepared as described by Chen and associates^30^. 100 ml of the bacterial culture for each strain was collected and disrupted by sonication, the unbroken cells and cell debris were removed by centrifugation at 14,000 *g* at 4 °C for 30 min. Supernatants were then centrifuged at 135,000 *g* at 4 °C for 1 h. The pellets, which contain membranes and ribosomes, were suspended in 1.0 ml cold TM buffer (10 mM Tris, pH 8.0, containing 8 mM MgSO_4_), and followed by centrifugation at 135,000 *g*. Pellets were then rinsed with 1.0 ml cold TM buffer, resuspended in 3.9 ml 0.25% (w/v) Sarkosyl and loaded into 3.9 ml ultracentrifuge tubes. After incubation at room temperature for 1 h, the tubes were centrifuged at 135,000 *g* for 1 h. Supernatants, containing the Sarkosyl-soluble inner membranes, were retained. The Sarkosyl-insoluble pellets, containing the outer membrane fraction, were washed twice with 1.0 ml 0.25% (w/v) Sarkosyl, incubated at room temperature for 1 h and centrifuged at 135,000 *g*. The pellets, containing the outer membrane fraction, were resuspended in 40 μl cold TM buffer.

The outer membrane proteins were also prepared as described by Leuzzi *et al*.^63^. Briefly, bacterial cells were disrupted by sonication and the supernatant containing the total membrane fraction was recovered and further centrifuged at 50,000 *g* for 90 min at 4 °C. The pellet containing the membranes was resuspended in 2% Sarkosyl in 20 mM Tris-HCl, pH 7.5 and 2 mM EDTA and incubated at room temperature to solubilize the inner membranes. To remove aggregates the suspension was first centrifuged at 10,000 *g* for 20 min at 4 °C and then centrifuged overnight at 75,000 *g* at 4 °C. The pellet containing the outer membranes was resuspended in SPB.

## Additional Information

**How to cite this article:** Li, L. *et al*. Identification of a novel type III secretion-associated outer membrane-bound protein from *Xanthomonas campestris* pv. *campestris. Sci. Rep.* **7**, 42724; doi: 10.1038/srep42724 (2017).

**Publisher's note:** Springer Nature remains neutral with regard to jurisdictional claims in published maps and institutional affiliations.

## Supplementary Material

Supplementary Information
