# Supplementary material for: Identification of a novel type III secretion-associated outer membrane-bound protein from Xanthomonas campestris pv. campestris
Source: Sci Rep. 2017 Feb 15;7:42724. doi: 10.1038/srep42724 (PMC5309889; doi:10.1038/srep42724)
Supplement: Supplementary Information [file srep42724-s1.pdf]

Supplementary information

Title: Identification of a novel type III secretion-associated outer membrane-bound protein from *Xanthomonas campestris* pv. *campestris*

Authors: Lei Li, Rui-Fang Li, Zhen-Hua Min, Guang-Tao Lu & Ji-Liang Tang

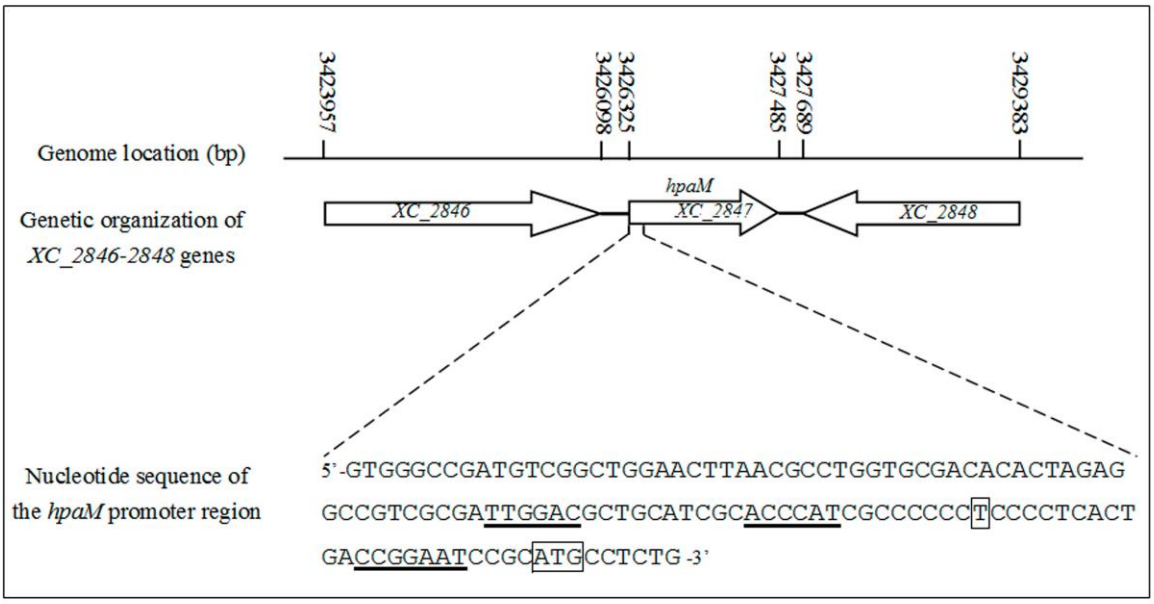

Supplementary Figure 1. Genetic and physical map of *hpaM* (*XC_2847*) gene in the genome of the *Xcc* strain 8004. The positions and orientations of the genes *XC_2846-2848* are shown: arrows indicate length, location and orientation of the genes; lines between arrows indicate intergenic sequences. The lower element shows 120 bp DNA senquence downstream of the previously annotated translational start codon (GTG) of *XC_2847*. The transcriptional start site determined in this study and the corresponding translational start codon (ATG) are indicated by square and rectangular frame, respectively. The putative -10 and -35 sequences and the Shine-Dalgarno sequence are shown underlined.

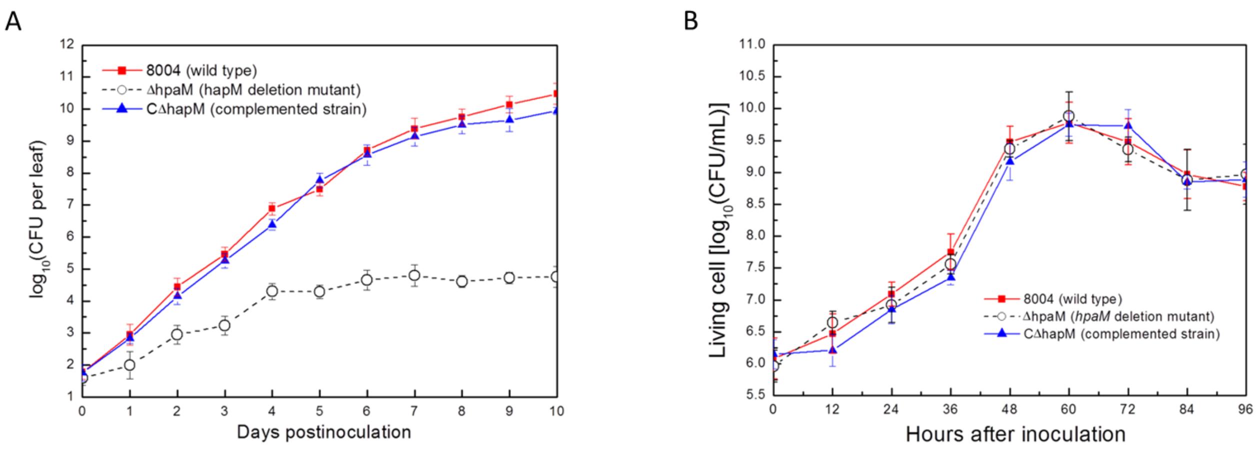

Supplementary Figure 2. Growth of *hpaM* mutant. A, Bacterial population of *Xcc* strains in the host plant Chinese radish leaves. Inoculated leaves for each strain were taken daily and homogenized in SPB (sodium phosphate buffer). The homogenates were diluted and plated on NYG plates. Bacterial CFUs were counted after incubation for 3 days. Data are the means and standard deviations from three replicates. B, Growth of *Xcc* strains in minimal medium MMX. Strains were inoculated into 100 ml MMX liquid medium. Samples were taken in triplicate at intervals of 12 h, diluted, and plated on NYG plates. Bacterial CFU were counted after incubation for 3 days.

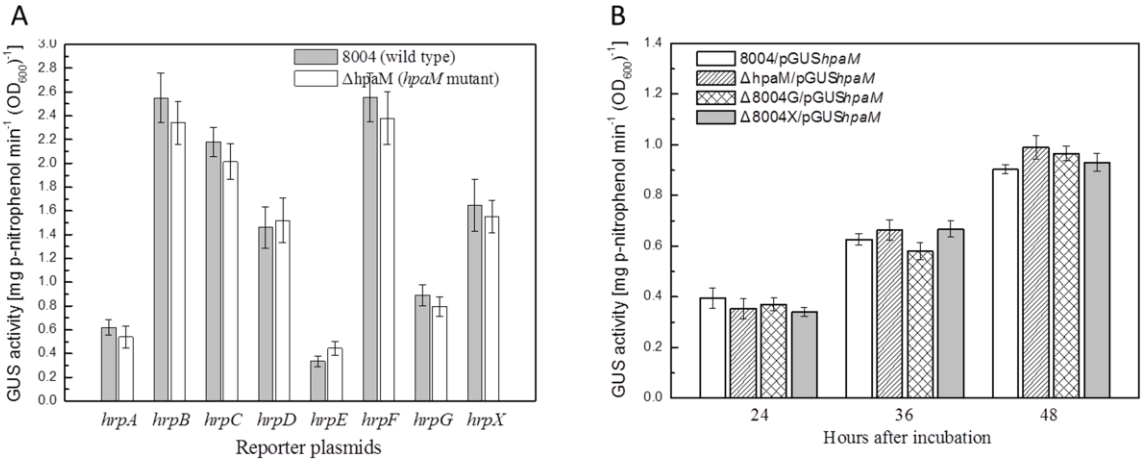

Supplementary Figure 3. No regulation between *hpaM* and *hrp* genes. **A,** GUS activity of *hrpG*, *hrpX* and six *hrp* operons (*hrpA*-*F*) promoter-*gusA* reporters in *hpaM* mutation and wild-type backgrounds. Strains were cultured in MMX medium for 48 h, and GUS activity was then determined by measurement of OD_{415} using ρ-nitrophenyl-β-D-glucuronide as substrate. Data are means ± standard deviations of triplicate measurements. The experiment was repeated twice, and similar results were obtained. **B,** GUS activity of *hpaM* promoter-*gusA* reporter in *hrpG*, *hrpX* mutation and wild-type backgrounds. Strains were cultured in MMX medium for 24, 36 and 48 h, and GUS activity in the cell lysate was determined. Data are means ± standard deviations of triplicate measurements. The experiment was repeated twice, and similar results were obtained.

A

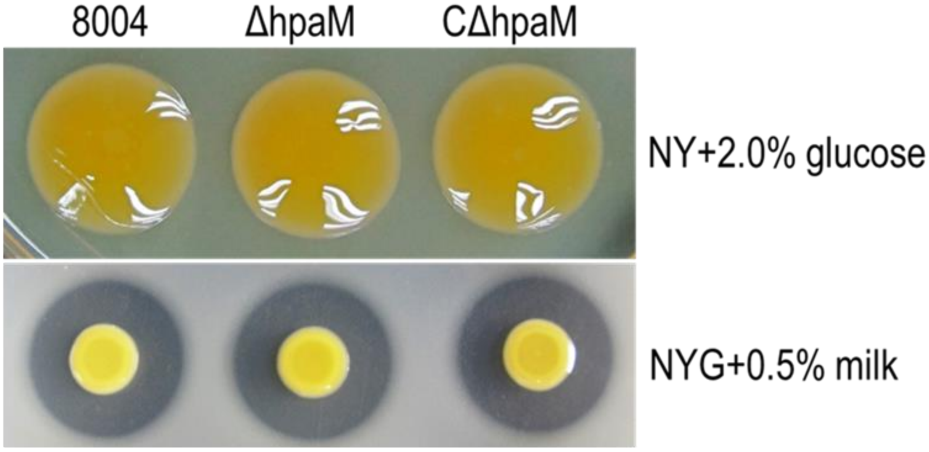

B

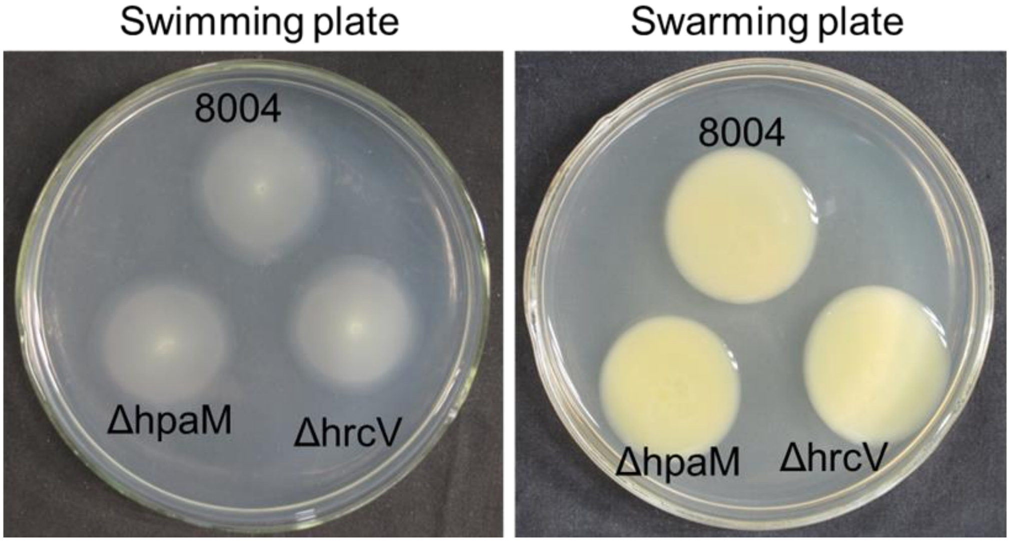

Supplementary Figure 4. HpaM mutation plays no impacts on the activity of extracellular proteases, EPS production and cell motility in *Xcc*. **A**, Plate assays (Tang et al. 1991) were used to test the activity of exoproteases, EPS production. An overnight culture (2 μl, OD_{600} = 1.0) of each *Xcc* strain was spotted onto a tested plate. For estimation of the activity of exoproteases, strains on NYG plates 0.5% (wt/vol) skim milk (Sangon, Shanghai, China) were incubated at 28 ^{o}C for 48 h. Zones of

clearance around the spot, which due to the degradation of the substrate, from each tested *Xcc* strain were similar, indicating the activity of exoproteases of *hpaM* mutant strain was similar to that of the wild type. For EPS production, bacteria on NY plates containing 2.0% (wt/vol) glucose were incubated at 28 ^{o}C for 5 days. The mutant displayed similar colonies to the wild-type strain, indicating the EPS yield of *hpaM* mutant strain was similar to that of the wild type strain. Similar results were obtained in two other independent experiments. **B**, Example photographs of *Xcc* wild type strain 8004, *hpaM* mutant strain ΔhpaM and *hrcV* mutant strain ΔhrcV on motility assay plates (Su et al., 2016). To detect swimming motility, an overnight culture (OD_{600} of 1.0) of each Xcc strain was stabbed into 0.28% agar plates composed of 0.03% Bacto peptone and 0.03% yeast extract followed by incubation at 28 oC for 4 days. To test swarming motility, the bacterial cells were inoculated onto NY plates containing 2% glucose and 0.6% agar using a toothpick, and then incubated at 28 ^{o}C for 3 days.

References

Su, H.Z., Wu, L., Qi, Y.H., Liu, G.F., Lu, G.T., Tang, J.L. (2016) Characterization of the GntR family regulator HpaR1 of the crucifer black rot pathogen *Xanthomonas campestris* pathovar *campestris*. *Sci Rep* **6**:19862.

Tang, J.L., Liu, Y.N., Barber, C.E., Dow, J.M., Wootton, J.C., Daniels, M.J. (1991) Genetic and molecular analysis of a cluster of rpf genes involved in positive regulation of synthesis of extracellular enzymes and polysaccharide in *Xanthomonas campestris* pathovar *campestris*. *Mol Gen Genet* **226**: 409–417.

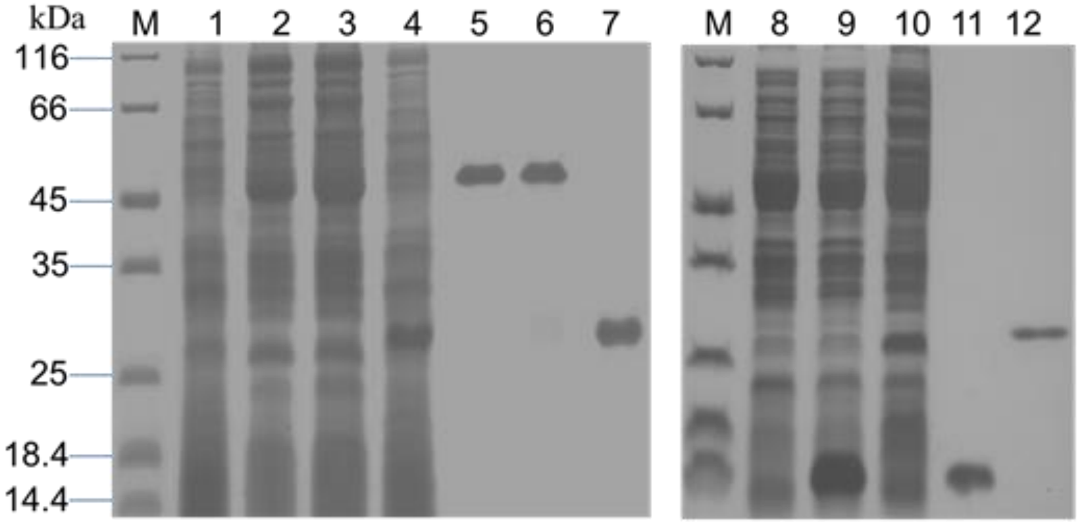

Supplementary Figure 5. His_6-tagged fusion proteins were overexpressed and purified. Lanes: 1, crude extract BL21/pET30a; 2, crude extract BL21/pET30a-$HpaM_{LN22}$ induced with IPTG; 3, crude extract BL21/pET30a-$HrcC_{34-370}$ induced with IPTG; 4, crude extract BL21/pET30a-$HrcJ_{22-206}$ induced with IPTG; 5, affinity-purified His_6-$HpaM_{LN22}$ protein; 6, affinity-purified His_6-$HrcC_{34-370}$ protein; 7, affinity-purified His_6-$HrcJ_{22-206}$ protein; 8, crude extract BL21/pET30a; 9, crude extract BL21/pET30a-$HpaM_{LN180-225}$ induced with IPTG; 10, crude extract BL21/pET30a-$HpaMLN_{180-320}$ induced with IPTG; 11, affinity-purified His_6-$HpaM_{LN180-225}$; 12, affinity-purified His_6-$HpaM_{LN180-320}$; M, molecular mass marker.

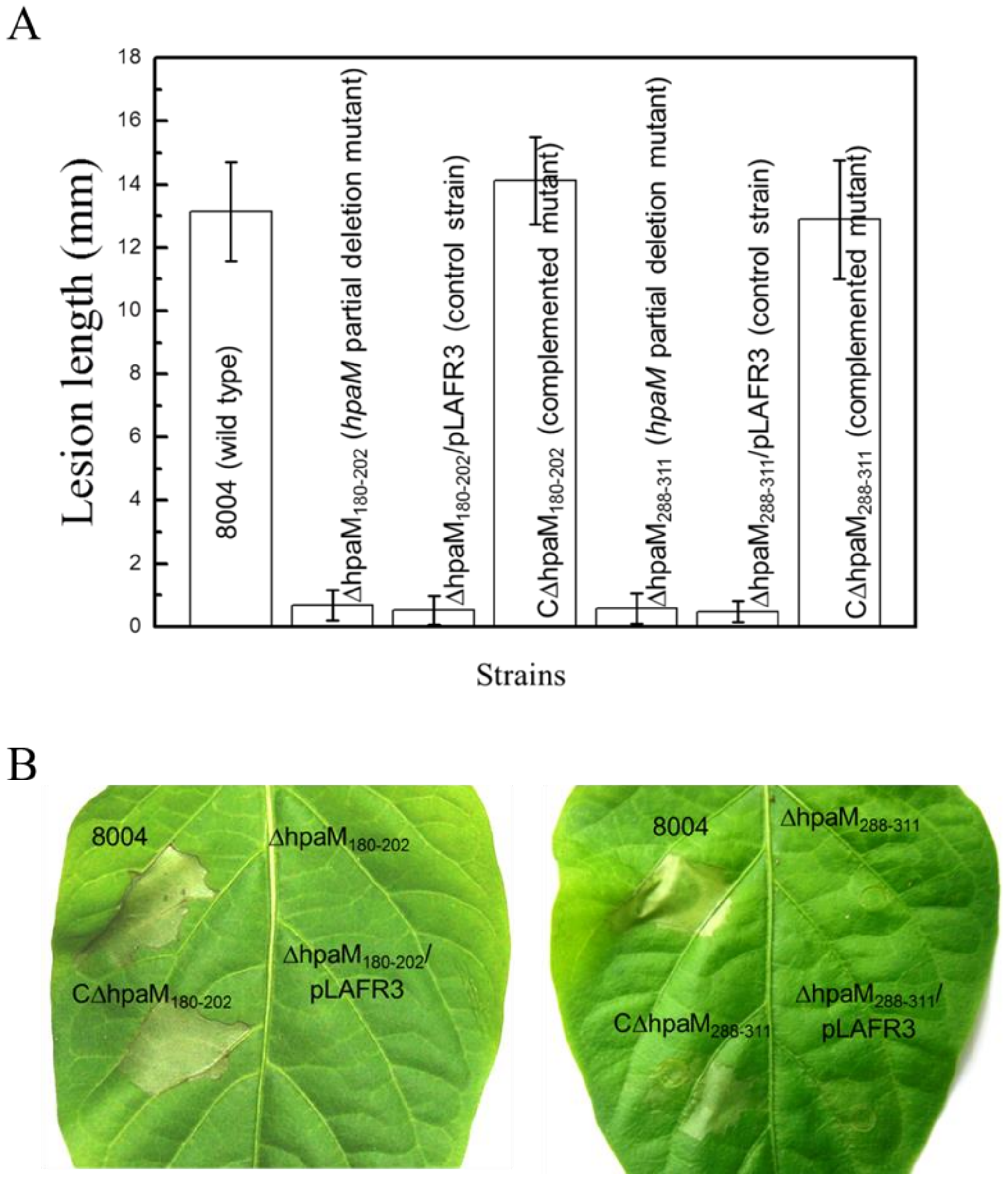

Supplementary Figure 6. *Xcc* mutant strain with HpaM deletion in 180th-202th aa (ΔhpaM$_{180\text{-}202}$) or 288th-311th aa (ΔhpaM$_{288\text{-}311}$) lose virulence in hose plant and the ability of HR induction in no-host plant. **A,** Mean lesion lengths caused by different strains in the host plant Chinese radish (*Raphanus sativus*). *Xcc* wild-type strain 8004 and its derivatives from overnight culture were washed and resuspended in SPB to an OD_{600} of 0.1. Leaves were cut with scissors dipped in the bacterial suspensions. The lesion lengths were measured at 10 days post-inoculation. Values given are the means and standard deviations from 15 measurements in one experiment. The experiment

was repeated three times with similar results. **B,** HR symptoms induced by *Xcc* strains in pepper leaves (*Capsicum annuum* cv. ECW-10R). Bacterial cells from overnight culture were washed with 10 mM SPB and resuspended in the same buffer to an OD_{600} of 0.1). Approximately 5 μl bacterial resuspension was infiltrated into the leaf mesophyll tissue with a blunt-end plastic syringe. Pictures of the inoculated pepper leaves were taken at 24 h after infiltration. The mutants $\Delta hpaM_{180\text{-}202}$ and $\Delta hpaM_{288\text{-}311}$ could not induce HR symptoms in pepper leaves. Three replications were done in each experiment, and each experiment was repeated three times. The results presented are from a representative experiment, and similar results were obtained in all other independent experiments.

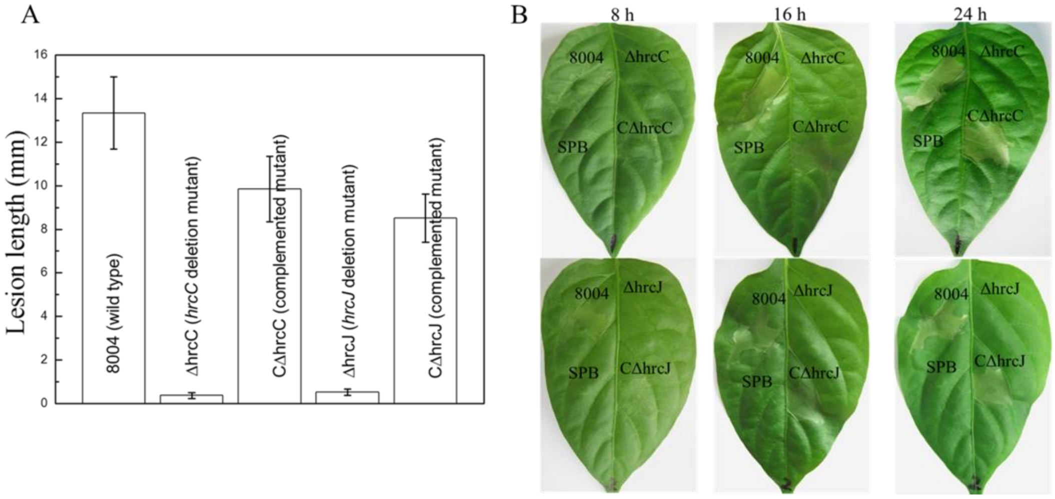

Supplementary Figure 7. HrcC and HrcJ are essential for pathogenicity of *Xcc*. **A,** the lesion length caused by *Xcc* wild-type strain 8004, *hrcC* deletion mutant ΔhrcC and its complemented strain CΔhrcC, *hrcJ* deletion mutant ΔhrcJ and its complemented strain CΔhrcJ. *Xcc* strains were inoculated into its host plant Chinese radish (*Raphanus sativus*) leaves and lesion lengths were scored 10 days postinoculation. Values represent means and standard deviation from twenty inoculated leaves in one experiment. The experiment was repeated three times with similar results. **B,** HR symptoms induced in pepper (*Capsicum annuum* cv. ECW-10R) leaves by *Xcc* strains. Bacterial cells from overnight culture were washed, resuspended in SPB (sodium phosphate buffer) to a concentration of OD_{600}=0.1 and infiltrated into the leaf mesophyll tissue. Pictures of the inoculated pepper leaves were taken at 8, 16 and 24 h after infiltration. Three replications were done in each experiment, and the experiment was repeated three times. The results presented are from a representative experiment, and similar results were obtained in all other independent experiments.

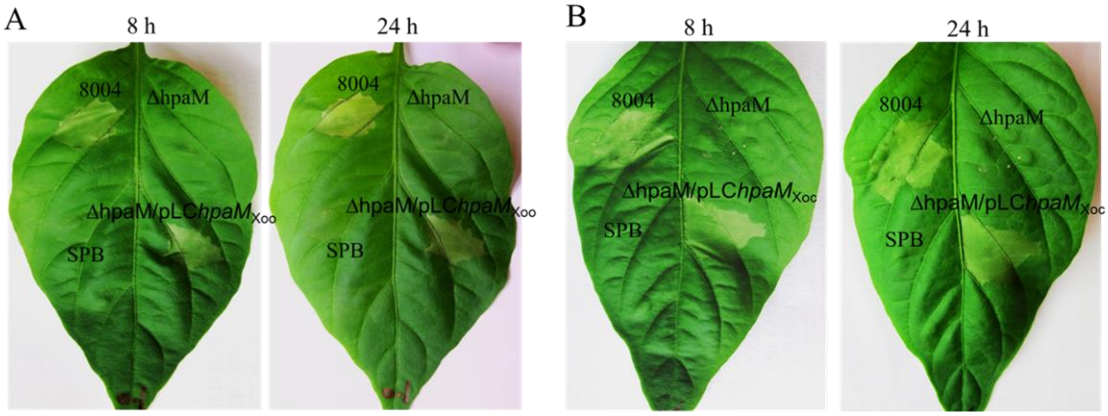

Supplementary Figure 8. Restoration of HR induction of *hpaM* mutant by *hpaM* homologues from *Xoo* (A) and *Xoc* (B). *Xcc* strains were cultured in NYG medium and resuspended in SPB to an OD_{600}=0.1 (1×10^{8} CFU ml^{-1}). Approximately 5 µl bacterial resuspension was infiltrated into the leaf mesophyll of pepper plant. The leaves were photographed at 8 and 24 h after infiltration. Three replications were done in each experiment, and the experiment was repeated three times. The results presented are from a representative experiment, and similar results were obtained in all other independent experiments. 8004, *Xcc* wild type strain; ΔhpaM, *Xcc hpaM* deletion mutant; ΔhpaM/pLC$hpaM_{Xoo}$, ΔhpaM harboring the recombinant plasmid that contains the *hpaM* homologue from *Xoo*; ΔhpaM/pLC$hpaM_{Xoc}$, ΔhpaM harboring the recombinant plasmid that contains the *hpaM* homologue from *Xoc.*

Table S1. Strains and plasmids used in this study

Strains or plasmids	Relevant characteristics	Reference or source
E. coli strains		
JM109	*RecA*1, *endA*1, *gyrA*96, *thi*, *supE*44, *relA*1 Δ (*lac-proAB*)/F' [*traD36*, *lacI*^{q}, *lacZ* ΔM15]	Yanisch-Perron *et al*., 1985
DH5α	Φ80Δ*lacZM*15 *recA1 endA1 deoR*	Gibco BRL, Life Technologies
BL21(DE3)	F$^-$ *ompT gal dcm lon hsdS*$_B$ (r^-_B m^-_B) λ(DE3)	Novagen, Germany
XL1-Blue MRF'	Reporter strain, Δ*(mcrA)183* Δ*(mcrCB-hsdSMR-mrr)173 endA1 hisB supE44 thi-1 recA1 gyrA96relA1 lac* [F ´*lacIq HIS3 aadA* Kan^{r}]	Stratagene
Xanthomonas oryzae pv. *oryzae* strains		
PXO99^A	Wild-type strain, Philippine race 6, Azacytidine resistant clone of PXO99, Rif^r	Hopkins *et al*., 1992
ΔhpaM$_{Xoo}$	*hpaM*$_{Xoo}$ gene (*PXO_01147*) deletion mutant derived from PXO99^A, Rif^r	This work
CΔhpaM$_{Xoo}$	ΔhpaM$_{Xoo}$ harboring pLC*hpaM*$_{Xoo}$, Rif^r Tet^r	This work
ΔhrcC$_{Xoo}$	*hrcC* gene deletion mutant derived from PXO99^A, Rif^r	Author's lab collection
ΔhpaM$_{Xoo}$/pR*hpaM*$_{Xoo}$H6	ΔhpaM$_{Xoo}$ harboring recombinant plasmid pR*hpaM*$_{Xoo}$H6, Rif^r Tet^r	This work
PXO99^A /pGUS*avrAC*	Wild-type strain PXO99^A harboring recombinant plasmid pGUS*avrAC*, Rif^r Tet^r	This work
ΔhpaM$_{Xoo}$/pGUS*avrAC*	ΔhpaM$_{Xoo}$ harboring recombinant plasmid pGUS*avrAC*, Rif^r Tet^r	This work
ΔhrcC$_{Xoo}$/pGUS*avrAC*	ΔhrcC$_{Xoo}$ harboring recombinant plasmid pGUS*avrAC*, Rif^r Tet^r	This work
Xanthomonas oryzae pv. *oryzicola* strains		
GX01	Wild-type strain, isolated from Guangxi, China, Rif^r	Author's lab collection
ΔhpaM$_{Xoc}$	*hpaM*$_{Xoc}$ gene deletion mutant derived from GX01, Rif^r	This study
CΔhpaM$_{Xoc}$	ΔhpaM$_{Xoc}$ harboring the plasmid pLC*hpaM*$_{Xoc}$, Rif^r Tet^r	This study
ΔhrcC$_{Xoc}$	*hrcC* gene deletion mutant derived from GX01, Rif^r	Author's lab collection
ΔhpaM$_{Xoc}$/pR*hpaM*$_{Xoc}$H6	ΔhpaM$_{Xoc}$ harboring the recombinant plasmid pR*hpaM*$_{Xoc}$H6, Rif^r Tet^r	This work
GX01/pGUS*avrAC*	GX01 harboring the recombinant plasmid	This work

	pGUS*avrAC*, Rif^r Tet^r	
ΔhpaM$_{Xoc}$/pGUS*avrAC*	ΔhpaM$_{Xoc}$ harboring the recombinant plasmid pGUS*avrAC*, Rif^r Tet^r	This work
ΔhrcC$_{Xoc}$/pGUS*avrAC*	ΔhrcC$_{Xoc}$ harboring the recombinant plasmid pGUS*avrAC*, Rif^r Tet^r	This work
Xanthomonas campestris pv. *campestris* strains		
8004	Wild-type strain, Rif^r	Daniels *et al*., 1984
083E12	As 8004, but *XC_2847*::Tn5*gusA*5, virulence-deficient mutant, Rif^r Kan^r	Author's lab collection
ΔhpaM	As 8004, but *hpaM* gene (*XC_2847*) deleted, non-polar effect. Rif^r	This work
CΔhpaM	ΔhpaM harboring the recombinant plasmid pLC*hpaM* , Rif^r Tet^r	This work
ΔhpaM/pLC*hpaM*$_{Xoo}$	ΔhpaM harboring the recombinant plasmid pLC*hpaM*$_{Xoo}$, Rif^r Tet^r	This work
ΔhpaM/pLC*hpaM*$_{Xoc}$	ΔhpaM harboring the recombinant plasmid pLC*hpaM*$_{Xoc}$, Rif^r Tet^r	This work
ΔhpaM$_{180-202}$	As 8004, but HpaM is deleted in 180th-202th aa, non-polar effect. Rif^r	This work
CΔhpaM$_{180-202}$	ΔhpaM$_{180-202}$ harboring the recombinant plasmid pLC*hpaM* , Rif^r Tet^r	This work
ΔhpaM$_{288-311}$	As 8004, but HpaM is deleted in 288th-311th aa, non-polar effect. Rif$^{r\ r}$	This work
CΔhpaM$_{288-311}$	ΔhpaM$_{288-311}$ harboring the recombinant plasmid pLC*hpaM* , Rif^r Tet^r	This work
ΔhpaM/pGUS*hpaM*	ΔhpaM harboring the recombinant plasmid pGUS*hpaM* , Rif^r Tet^r	This work
ΔavrBs1$_{Xcc}$	As 8004, but the avirulence gene *avrBs1*$_{Xcc}$ (*XC_2081*) deleted, non-polar effect. Rif^r	Xu *et al*., 2008
ΔhpaM/pR*hpaM*H6	ΔhpaM harboring the recombinant plasmid pR*hpaM*H6, Rif^r Tet^r	This work
8004/pGUS*avrAC*	8004 harboring the reporter plasmid pGUS*avrAC*, Rif^r Tet^r	This work
8004/pGUS*xopN*	8004 harboring the reporter plasmid pGUS*xopN*, Rif^r Tet^r	This work
8004/pL6*gus*	8004 harboring the recombinant plasmid pL6*gus*, Rif^r Tet^r	This work
8004/pL*avrAC*$_{102}$::CyaA	8004 harboring the reporter plasmid pL*avrAC*$_{102}$::CyaA, Rif^r Tet^r	This work
ΔhpaM/pGUS*avrAC*	ΔhpaM harboring the reporter plasmid pGUS*avrAC*, Rif^r Tet^r	This work

ΔhpaM/pGUS*xopN*	ΔhpaM harboring the reporter plasmid pGUS*xopN*, Rif^r Tet^r	This work
ΔhpaM/pL*avrAC*$_{102}$::CyaA	ΔhpaM harboring the reporter plasmid pL*avrAC*$_{102}$::CyaA, Rif^r Tet^r	This work
Δ8004G	As 8004, but *hrpG* deleted, Rif^r Kan^r	Author's lab collection
Δ8004X	As 8004, but *hrpX* deleted, Rif^r Kan^r	Author's lab collection
ΔhrcV	As 8004, but *hrcV* deleted, Rif^r	Author's lab collection
ΔhrcV/pGUS*avrAC*	ΔhrcV harboring the reporter plasmid pGUS*avrAC*, Rif^r Tet^r	This work
ΔhrcV/pGUS*xopN*	ΔhrcV harboring the reporter plasmid pGUS*xopN*, Rif^r Tet^r	This work
ΔhrcV/pL*avrAC*$_{102}$::CyaA	ΔhrcV harboring the reporter plasmid pL*avrAC*$_{102}$::CyaA, Rif^r Tet^r	This work
ΔhrcC	As 8004, but *hrcC* deleted, Rif^r	Author's lab collection
ΔhrcC/pR*hrcC*H6	ΔhrcC harboring the recombinant plasmid pR*hrcC*H6, Rif^r Tet^r	This work
ΔhrcJ	As 8004, but *hrcJ* deleted, Rif^r	Author's lab collection
ΔhrcJ/pR*hrcJ*H6	ΔhrcJ harboring the recombinant plasmid pR*hrcJ*H6, Rif^r Tet^r	This work
ΔhpaR1/pR*hpaR1*H6	ΔhpaR1 harboring the recombinant plasmid pR*hpaR1*H6, Rif^r Tet^r	Author's lab collection
ΔhpaS/pR*hpaS*H6	ΔhpaS harboring the recombinant plasmid pR*hpaS*H6, Rif^r Tet^r	Author's lab collection
ΔhpaM-hrcC	As 8004, but both *hpaM* and *hrcC* deleted, Rif^r	This work
ΔhpaM-hrcC/pR*hpaM*H6	ΔhpaM-HrcC harboring the recombinant plasmid pR*hpaM*H6, Rif^r Tet^r	This work
ΔavrAC	As 8004, but *avrAC* gene (*XC_1553*) deleted, Rif^r	This work
ΔavrAC-hpaM	As 8004, but both *avrAC* and *hpaM* deleted, Rif^r	This work
ΔavrAC-hrcV	As 8004, but both *avrAC* and *hrcV* deleted, Rif^r	This work
ΔavrAC/pR*avrAC*H6	ΔavrAC harboring the recombinant plasmid pR*avrAC*H6, Rif^r Tet^r	This work
ΔavrAC-hpaM/pR*avrAC*H6	ΔavrAC-hpaM harboring the recombinant plasmid pR*avrAC*H6, Rif^r Tet^r	This work
ΔavrAC-hrcV/pR*avrAC*H6	ΔavrAC-hrcV harboring the recombinant	This work

	plasmid pR*avrAC*H6, Rif^r Tet^r	
8004/pGUS*hpaM*	8004 harbouring the recombinant plasmid pGUS*hpaM*, Rif^r Tet^r	This work
8004/pGUS*hrpA*	8004 harbouring the recombinant plasmid pGUS*hrpA*, Rif^r Tet^r	This work
8004/pGUS*hrpB*	8004 harbouring the recombinant plasmid pGUS*hrpB*, Rif^r Tet^r	This work
8004/pGUS*hrpC*	8004 harbouring the recombinant plasmid pGUS*hrpC*, Rif^r Tet^r	This work
8004/pGUS*hrpD*	8004 harbouring the recombinant plasmid pGUS*hrpD*, Rif^r Tet^r	This work
8004/pGUS*hrpE*	8004 harbouring the recombinant plasmid pGUS*hrpE*, Rif^r Tet^r	This work
8004/pGUS*hrpF*	8004 harbouring the recombinant plasmid pGUS*hrpF*, Rif^r Tet^r	This work
8004/pGUS*hrpG*	8004 harbouring the recombinant plasmid pGUS*hrpG*, Rif^r Tet^r	This work
8004/pGUS*hrpX*	8004 harbouring the recombinant plasmid pGUS*hrpX*, Rif^r Tet^r	This work
Δ8004G/pGUS*hpaM*	Δ8004G harbouring the recombinant plasmid pGUS*hpaM*, Rif^r Kan^r Tet^r	This work
Δ8004X/pGUS*hpaM*	Δ8004X harbouring the recombinant plasmid pGUS*hpaM*, Rif^r Kan^r Tet^r	This work
8004 /pLAFR3	8004 harbouring the plasmid pLAFR3, Rif^r Tet^r	This work
ΔhpaM/pGUS*hpaM*	ΔhpaM harbouring the recombinant plasmid pGUS*hpaM*, Rif^r Tet^r	This work
ΔhpaM/pGUS*hrpA*	ΔhpaM harbouring the recombinant plasmid pGUS*hrpA*, Rif^r Tet^r	This work
ΔhpaM/pGUS*hrpB*	ΔhpaM harbouring the recombinant plasmid pGUS*hrpB*, Rif^r Tet^r	This work
ΔhpaM/pGUS*hrpC*	ΔhpaM harbouring the recombinant plasmid pGUS*hrpC*, Rif^r Tet^r	This work
ΔhpaM/pGUS*hrpD*	ΔhpaM harbouring the recombinant plasmid pGUS*hrpD*, Rif^r Tet^r	This work
ΔhpaM/pGUS*hrpE*	ΔhpaM harbouring the recombinant plasmid pGUS*hrpE*, Rif^r Kan^r Tet^r	This work
ΔhpaM/pGUS*hrpF*	ΔhpaM harbouring the recombinant plasmid pGUS*hrpF*, Rif^r Tet^r	This work
ΔhpaM/pGUS*hrpG*	ΔhpaM harbouring the recombinant plasmid pGUS*hrpG*, Rif^r Tet^r	This work
ΔhpaM/pGUS*hrpX*	ΔhpaM harbouring the recombinant plasmid pGUS*hrpX*, Rif^r Tet^r	This work

ΔhpaM/pLAFR3	ΔhpaM harbouring the plasmid pLAFR3, Rif^r Tet^r	This work
Plasmids		
pLAFR3	Broad host range cloning vector, Tet^r	Staskawicz *et al*., 1987
pLAFR6	A promoterless derivative of pLAFR3, Tet^r	Huynh *et al*., 1989
pRK2073	Helper plasmid, Tra$^+$, Mob$^+$, ColE1, Spc^r.	Leong *et al*., 1982
pK18*mobsacB*	pUC18 derivative, *lacZα*, *sacB*, Kan^r, *mob* site. Allelic exchange vector (Suicidal vector carrying *sacB* gene for mutagenesis)	Schäfer *et al*., 1994
pET-30a	Expression vector, allow the production of fusion proteins containing amino terminal 6×His-tagged sequences. Kan^r	Novagen
pET-30a-HpaM$_{LN22}$	pET-30a containing a 981-bp fragment of truncated *hpaM* gene encoding the 23th–349th amino acids.	This work
pET-30a-HpaM$_{LN180-225}$	pET-30a containing a 138-bp fragment of truncated *hpaM* gene encoding the 180th–225th amino acids.	This work
pET-30a-HpaM$_{LN180-320}$	pET-30a containing a 423-bp fragment of truncated *hpaM* gene encoding the 180th–320th amino acids.	This work
pET-30a-HrpC$_{34-370}$	pET-30a containing a 1011-bp fragment of partial *hrcC* gene sequence encoding the 34th–370th amino acids.	This work
pET-30a-HrcJ$_{22-206}$	pET-30a containing a 555-bp fragment of partial *hrcJ* gene sequence encoding the 22th -206th amino acids.	This work
pLC*hpaM*	1432-bp DNA fragment containing the full *hpaM* gene (*XC_2847*) of *Xcc* strain cloned into the plasmid pLAFR6. Tet^r	This work
pLC*hpaM*$_{Xoo}$	1053-bp DNA fragment of the *hpaM*$_{Xoo}$ gene ORF (*PXO_01147*) of *Xoo* strain cloned into the plasmid pLAFR3. Tet^r	This work
pLC*hpaM*$_{Xoc}$	1053-bp DNA fragment of the *hpaM*$_{Xoc}$ gene ORF (*XOC3053*) of *Xoc* strain cloned into the plasmid pLAFR3. Tet^r	This work
pBT	Two-hybrid system bait plasmid containing the *cat* gene, p15A origin of replication and λ cI ORF	Stratagene
pB*hpaM*$_{LN22}$	pBT derivative carrying the 981-bp of *hpaM* gene lacking the nt 1 to 66, Cat^r	This work

pB*hrcC*$_{34\text{-}370}$	pBT derivative carrying a 1011-bp fragment of partial *hrcC* gene sequence encoding the 34th–370th amino acids, Cat^{r}	This work
pBT*hpaS*$_{LN54}$	pBT derivative carrying *hpaS* gene	Li *et al*., 2014
pTRG	Two-hybrid system target plasmid containing the *tet* gene, ColE1 origin of replication, and RNA polymerase α subunit ORF	Stratagene
pT*hrcC*$_{LN33}$	pTRG derivative carrying the 1716-bp of truncated *hrcC* gene lacking the nt 1 to 99, Tet^{r}	This work
pT*hrcC*$_{34\text{-}370}$	pTRG derivative carrying 1011-bp fragment of partial *hrcC* gene sequence encoding the 34th–370th amino acids, Tet^{r}	This work
pT*hrcJ*$_{LN21}$	pTRG derivative carrying 699-bp of truncated *hrcJ* gene lacking the nt 1 to 63, Tet^{r}	This work
pT*hrcJ*$_{22\text{-}206}$	pTRG derivative carrying 555-bp truncated *hrcJ* gene sequence encoding the 22th–206th amino acids, Tet^{r}	This work
pTRG*hrpG*	pTRG derivative carrying the full length of the coding region of *hrpG* gene (789-bp), Tet^{r}	Li *et al*., 2014
pTRG*hpaS*$_{LN54}$	pTRG derivative carrying truncated *hpaS* gene lacking the nt 1–162 , Tet^{r}	Author’s lab collection
pBM$_{23\text{-}202}$	pBT derivative carrying a 540-bp fragment encoding the 23th–202th amino acids of HpaM, Cat^{r}	This work
pBM$_{23\text{-}225}$	pBT derivative carrying a 609-bp fragment encoding the 23th–225th amino acids of HpaM, Cat^{r}	This work
pBM$_{23\text{-}248}$	pBT derivative carrying a 678-bp fragment encoding the 23th–248th amino acids of HpaM, Cat^{r}	This work
pBM$_{226\text{-}349}$	pBT derivative carrying a 372-bp fragment encoding the 226th–349th amino acids of HpaM, Cat^{r}	This work
pBM$_{203\text{-}349}$	pBT derivative carrying a 441-bp fragment encoding the 203th–349th amino acids of HpaM, Cat^{r}	This work
pBM$_{180\text{-}349}$	pBT derivatives carrying the 510-bp fragment encoding 180th–349th amino acids of HpaM, Cat^{r}	This work
pBM$_{180\text{-}271}$	pBT derivative carrying a 276-bp fragment encoding the 180th–271th amino acids of HpaM, Cat^{r}	This work

$pBM_{180\text{-}225}$	pBT derivative carrying a 138-bp fragment encoding the 180th–225th amino acids of HpaM, Cat^r	This work
$pBM_{180\text{-}300}$	pBT derivative carrying a 363-bp fragment encoding the 180th–300th amino acids of HpaM, Cat^r	This work
$pBM_{180\text{-}320}$	pBT derivative carrying a 423-bp fragment encoding the 180th–320th amino acids of HpaM, Cat^r	This work
$pBM_{180\text{-}340}$	pBT derivative carrying a 483-bp fragment encoding the 180th–340th amino acids of HpaM, Cat^r	This work
pL6*gus*	pLAFR6 containing a 1,832-bp *gusA* ORF (excluding the translational start codon ATG), Tet^r	Jiang *et al*., 2008
pGUS*hpaM*	pLAFR6 containing 404-bp promoter region of the gene *hpaM* fused to the coding region for *gusA*, Tet^r	This work
pGUS*hrpG*	pLAFR6 containing an *hrpG* promoter-*gusA* fusion fragment, Tet^r	Author's lab collection
pGUS*hrpX*	pLAFR6 containing an *hrpX* promoter-*gusA* fusion fragment, Tet^r	Author's lab collection
pGUS*hrpA*	pLAFR6 containing an *hrpA* operon promoter-*gusA* fusion fragment, Tet^r	Author's lab collection
pGUS*hrpB*	pLAFR6 containing an *hrpB* operon promoter-*gusA* fusion fragment, Tet^r	Author's lab collection
pGUS*hrpC*	pLAFR6 containing an *hrpC* operon promoter-*gusA* fusion fragment, Tet^r	Author's lab collection
pGUS*hrpD*	pLAFR6 containing an *hrpD* operon promoter-*gusA* fusion fragment, Tet^r	Author's lab collection
pGUS*hrpE*	pLAFR6 containing an *hrpE* operon promoter-*gusA* fusion fragment, Tet^r	Author's lab collection
pGUS*hrpF*	pLAFR6 containing an *hrpF* operon promoter-*gusA* fusion fragment, Tet^r	Author's lab collection
pGUS*xopN*	pLAFR6 containing the type III secretion signal of *xopN* (from the 488th bp upstream to the 159th bp downstream of the start codon of *XC_0241*) encoding sequence fused with promoterless *gus* gene, Tet^r	Author's lab collection
pGUS*avrAC*	pLAFR6 containing the type III secretion signal of AvrAC (from the 400th bp upstream to the 600th bp downstream of the start codon of *XC_1553*) encoding sequence fused with promoterless *gus* gene, Tet^r	Author's lab collection

pL*avrAC*$_{102}$::CyaA	pLAFR6 containing the type III secretion signal coding sequence of AvrAC (from the 588th bp upstream to the 306th bp downstream of the start codon) fused with *cyaA* gene (excluding start codon ATG), Tet^{r}	Granted by Prof. Jiang
pR*hpaM*H6	pLAFR3 containing the encoding sequence of HpaM with 6×His tag in its C-terminus, Tet^{r}	This work
pR*hpaM*$_{Xoo}$H6	pLAFR3 containing the encoding sequence of HpaM$_{Xoo}$ with 6×His tag in its C-terminus, Tet^{r}	This work
pR*hpaM*$_{Xoc}$H6	pLAFR3 containing the encoding sequence of HpaM$_{Xoc}$ with 6×His tag in its C-terminus, Tet^{r}	This work
pR*avrAC*H6	pLAFR6 containing the encoding sequence of AvrAC with 6xHis tag in its C-terminus , Tet^{r}	This work
pR*hpaR1*H6	pLAFR3 containing the encoding sequence of HpaR1 with 6×His tag in its C-terminus, Tet^{r}	Author's lab collection
pR*hpaS*H6	pLAFR3 containing the encoding sequence of HpaS with 6×His tag in its C-terminus, Tet^{r}	Author's lab collection
pR*hrcC*H6	pLAFR3 containing the encoding sequence of HrcC with 6×His tag in its C-terminus, Tet^{r}	This work
pR*hrcJ*H6	pLAFR3 containing the encoding sequence of HrcJ with 6×His tag in its C-terminus, Tet^{r}	This work

References

Daniels, M.J., Barber, C.E., Turner, P.C., Sawczyc, M.K., Byrde, R.J.W., and Fielding, A.H. (1984) Cloning of genes involved in pathogenicity of *Xanthomonas campestris* pv. *campestris* using the broad host range cosmid pLAFR1. *EMBO J* **3**: 3323–3328.

Hopkins, C.M., White, F.F., Choi, S.H., Guo, A., and Leach, J.E. (1992) Identification of a family of avirulence genes from *Xanthomonas oryzae* pv. *oryzae*. *Mol Plant Microbe Interact* **5**: 451–459.

Huynh, T.V., Dahlbeck, D., and Staskawicz, B.J. (1989) Bacterial blight of soybean: regulation of a pathogen gene determining host cultivar specificity. *Science* **245**: 1374–1377.

Jiang, B.L., He, Y.Q., Cen, W.J., Wei, H.Y., Jiang, G.F., Jiang, W., *et al*. (2008) The type III secretion effector XopXccN of *Xanthomonas campestris* pv. *campestris* is required for full virulence. *Res Microbiol* **159**: 216–220.

Leong, S.A., Ditta, G.S., and Helinski, D.R. (1982) Heme biosynthesis in *Rhizobium*. Identification of a cloned gene coding for delta-aminolevulinic acid synthetase from *Rhizobium meliloti*. *J Biol Chem* **257**: 8724–8730.

Li, R.F., Lu, G.T., Li, L., Su, H.Z., Feng, G.F., Chen, Y., *et al*. (2014) Identification of a putative cognate sensor kinase for the two-component response regulator HrpG, a key regulator controlling the expression of the *hrp* genes in *Xanthomonas campestris* pv. *campestris*. *Environ Microbiol* **16**: 2053–2071.

Schäfer, A., Tauch, A., Jäger, W., Kalinowski, J., Thierbach, G., and Pühler, A. (1994) Small mobilizable multi-purpose cloning vectors derived from the *Escherichia coli* plasmids pK18 and pK19: selection of defined deletions in the chromosome of *Corynebacterium glutamicum*. *Gene* **145**: 69–73.

Staskawicz, B., Dahlbeck, D., Keen, N., and Napoli, C. (1987) Molecular characterization of cloned avirulence genes fromrace 0 and race 1 of *Pseudomonas syringae* pv. *glycinea*. *J Bacteriol* **169**: 5789–5794.

Xu, R.Q., Blanvillain, S., Feng, J.X., Jiang, B.L., Li, X.Z., Wei, H.Y., *et al*. (2008) $AvrAC_{(Xcc8004)}$, a type III effector with a leucine-rich repeat domain from *Xanthomonas campestris* pathovar *campestris* confers avirulence in vascular tissues of Arabidopsis thaliana ecotype Col-0. *J Bacteriol* **190**: 343–55.

Yanisch-Perron, C., Vieira, J., and Messing, J. (1985) Improved M13 phage cloning vectors and host strains: nucleotide sequences of the M13mp18 and pUC19 vectors. *Gene* **33**: 103–119.

Table S2. Primers used in this study §

Primer	Nucleotide sequence (5′→3′)	The amplified fragment or the utilization
L*hpaM*-F<br>L*hpaM*-R	GACGAATTCGATGGCGTCAACCAGCTCTC<br>GCGTCTAGATCTAGTGTGTCGCACCAGGC	747-bp DNA sequence upstream of *hpaM*, used for construction of *hpaM* deletion mutant.
R*hpaM*-F<br>R*hpaM*-R	GACTCTAGAGATGTGGCCGGCGTGGATATC<br>GACAAGCTTATTCCTGGACGTGGCGATGC	726-bp DNA sequence downstream of *hpaM*, used for construction of *hpaM* deletion mutant.
L*hpaM*-F_{180}<br>L*hpaM*-R_{180}	ACAGTTGGATCC CTACCTGATCATCAATCG<br>ACAGTTTCTAGA GTCGTATTCGCTGAGCAG	767-bp fragment spans 230th nt upstream to 537th nt downstream of the start codon ATG of *hpaM* ORF, used for construction of HpaM derivative which deleted in 180th-202th aa.
L*hpaM*-F_{180}<br>L*hpaM*-R_{180}	ACAGTTTCTAGA ACCGCCAAGGTGACGATG<br>ACAGTTAAGCTT CAGCAACCACCAGGTAGG	560-bp fragment spans 607th nt to 1166th nt downstream of the start codon ATG of *hpaM* ORF , used for construction of HpaM derivative which deleted in 180th-202th aa a.
L*hpaM*-F_{311}<br>L*hpaM*-R_{311}	ACAGTTGGATCC AAAAGCCATCGACAAGCT<br>ACAGTTTCTAGA CATGCTGTTGTCCCAGCACA	715-bp DNA fragment spans 147th nt to 861th nt downstream of the start codon ATG of *hpaM* ORF, used for construction of HpaM derivative which deleted in 288th-311th aa.
L*hpaM*-F_{311}<br>L*hpaM*-R_{311}	ACAGTTTCTAGA GCGGCCAACTGCAGCGCGGCC<br>ACAGTTAAGCTT TGCCGCCGCGCACGTGAG	588-bp DNA fragment spans 934th nt to 1521th nt downstream of the start codon ATG of *hpaM* ORF , used for construction of HpaM derivative which deleted in 288th-311th aa.
C*hpaM*-F<br>C*hpaM*-R	ACAGTTGGATCCGCCTACAGCTTCTGAGCCTG<br>ACAGTTAAGCTTGTGGCTGCGTAGCGGTTTTG	1432-bp DNA fragment spans 352 upstream to 30 bp downstream of the *hpaM* ORF sequence.
hpaM-F<br>*hpaM*-R	ACAGTTGGATCCATGCCTCTGCTTGCCCGCTC<br>ACAGTTAAGCTTTTAGTGGTGGTGGTGGTGGTGCTCGTGGC GCACGCA	DNA fragment of 1047-bp *hpaM* coding sequence fusing with 6×His tag encoding sequences. Used for Western blot analysis.
hpaM-1F<br>*hpaM*-1R	GATGGATCCGCACCGCCAGCGCCTGCC<br>GGGCTCGAGTTACTCGTGGCGCACGCA	981-bp DNA fragment spans nucleotides 67 to 1047 bp of the *hpaM* coding sequence, encoding the 23th-349th amino acids. Used for bacterial two-hybrid assay.
hpaM-2F<br>*hpaM*-2R	GATGGGATCCGCACCGCCAGCGCCTGCC<br>GCGGCTCGAGGCGATTGAACGACACGCC	540-bp DNA fragment spans nucleotides 67 to 606 bp of the *hpaM* ORF sequence, encoding the 23th-202th amino acids. Used for bacterial two-hybrid assay.
hpaM-3F<br>*hpaM*-3R	GATGGGATCCGCACCGCCAGCGCCTGCC<br>GCGGCTCGAGATGCTCGGTGGTGAGGCC	609-bp DNA fragment spans nucleotides 67 to 675 bp of the *hpaM* ORF sequence,

		encoding the 23th-225th amino acids. Used for bacterial two-hybrid assay.
hpaM-4F<br>*hpaM*-4R	GATGGGATCCGCACCGCCAGCGCCTGCC<br>GGGGCTCGAGATCCGACAGATAGATGCC	678-bp DNA fragment spans nucleotides 67 to 744 bp of the *hpaM* ORF sequence, encoding the 23th-248th amino acids. Used for bacterial two-hybrid assay.
hpaM-5F<br>*hpaM*-5R	GAGGGATCCCTGGAAGACAGCGAGATTCG<br>GGGCTCGAGTTACTCGTGGCGCACGCA	372-bp DNA fragment spans nucleotides 676 to 1047 bp of the *hpaM* ORF sequence, encoding the 226th-349th amino acids. Used for bacterial two-hybrid assay.
hpaM-6F<br>*hpaM*-6R	GGGGGATCCACCGCCAAGGTGACGATGATG<br>GGGCTCGAGTTACTCGTGGCGCACGCA	441-bp DNA fragment spans nucleotides 607 to 1047 bp of the *hpaM* ORF sequence, encoding the 203th-349th amino acids. Used for bacterial two-hybrid assay.
hpaM-7F<br>*hpaM*-7R	GGGGGATCCACGCGCGAGATCACCATC<br>GGGCTCGAGTTACTCGTGGCGCACGCA	510-bp DNA fragment spans nucleotides 538 to 1047 bp of the *hpaM* ORF sequence, encoding the 180th-349th amino acids. Used for bacterial two-hybrid assay.
hpaM-8F<br>*hpaM*-8R	GGGGGATCC ACGCGCGAGATCACCATC<br>GGGCTCGAG GCATGCCAGGAAGATGCC	276-bp DNA fragment spans nucleotides 538 to 813 bp of the *hpaM* ORF sequence, encoding the 180th-271th amino acids. Used for bacterial two-hybrid assay.
hpaM-9F<br>*hpaM*-9R	GGGGGATCCACGCGCGAGATCACCATC<br>GGGCTCGAGATGCTCGGTGGTGAGGCC	138-bp DNA fragment spans nucleotides 538 to 675 bp of the *hpaM* ORF sequence, encoding the 180th-225th amino acids. Used for bacterial two-hybrid assay.
*hpaM*O-9F<br>*hpaM*O-9R	ACAGTTGGATCCACGCGCGAGATCACCATCG<br>ACAGTTAAGCTT ATGCTCGGTGGTGAGGCC	138-bp DNA fragment spans nucleotides 538 to 675 bp of the *hpaM* ORF sequence, encoding 180th-225th amino acids. Used for overexpression and pull-down assay.
hpaM-10F<br>*hpaM*-10R	GGGGGATCCACGCGCGAGATCACCATC<br>GGGCTCGAG GCTGGCGAAACGGTTGTT 300	363-bp DNA fragment spans nucleotides 538 to 900 bp of the *hpaM* ORF sequence, encoding the 180th-300th amino acids. Used for bacterial two-hybrid assay.
hpaM-11F<br>*hpaM*-11R	GGGGGATCCACGCGCGAGATCACCATC<br>GGGCTCGAG GAAGTCGGCCGCGCTGCA 320	423-bp DNA fragment spans nucleotides 538 to 960 bp of the *hpaM* ORF sequence, encoding the 180th-320th amino acids. Used for bacterial two-hybrid assay.
*hpaM*O-11F<br>*hpaM*O-11R	ACAGTTGGATCCACGCGCGAGATCACCATCG<br>ACAGTTAAGCTTGAAGTCGGCCGCGCTGCA	423-bp DNA fragment spans nucleotides 538 to 960 bp of the *hpaM* ORF sequence, encoding the 180th-320th amino acids. Used for overexpression and pull-down assay.
hpaM-12F	GGGGGATCCACGCGCGAGATCACCATC	483-bp DNA fragment spans nucleotides

hpaM-12R	GGGCTCGAGCTGCGGCTCGATATCCAC 340	538 to 1020 bp of the *hpaM* ORF sequence, encoding the 180th-340th amino acids. Used for bacterial two-hybrid assay.
hpaM-OF<br>*hpaM*-OR	GGGGATCCGCACCGCCAGCGCCTGCC<br>GGGAAGCTTTTACTCGTGGCGCACGCA	981-bp DNA fragment spans nucleotides 67 to 1047 bp of the *hpaM* gene sequence, encoding the 23th-349th amino acids. Used for overexpression and pull-down assay.
hrcC-F<br>*hrcC*-R	GATTGGATCCGCCGCGTCGGTGCCGTGG<br>GGGCTCGAGTCAGGGCGAGACGATATG	1716-bp DNA fragment spans nucleotides 100 to 1815 bp of *hrcC* ORF, encoding the 34th-605th amino acids. Used for bacterial two-hybrid assay.
hrcC-N1F<br>*hrcC*-N1R	GATTGGATCCGCCGCGTCGGTGCCGTGG<br>GGGCTCGAGATCGATCTGCAGCAGCTT	1011-bp DNA fragment spans nucleotides 100 to 1110 bp of the *hrcC* ORF sequence, encoding the 34th-370th amino acids. Used for bacterial two-hybrid assay.
hrcC-N2F<br>*hrcC*-N2R	GGGGATCCGCCGCGTCGGTGCCGTGG<br>GGGAAGCTTATCGATCTGCAGCAGCTT	1011-bp DNA fragment spans nucleotides 100 to 1110 bp of the *hrcC* ORF sequence, encoding the 34th-370th amino acids. Used for overexpression and pull-down assay.
hrcJ-F<br>*hrcJ*-R	GGGGGATCCCAGCTGTATTCCGGGCTGAC<br>GGGCTCGAGTCACCCCGCATGTTTTCT	699-bp DNA fragment spans nucleotides 64 to 762 bp of *hrcJ* gene sequence, encoding the 22th-254th amino acids. Used for bacterial two-hybrid assay.
hrcJ-N1F<br>*hrcJ*-N1R	GGGGGATCCCAGCTGTATTCCGGGCTGAC<br>GATTCTCGAG GGCACGCGGCGGCGCCGA	555-bp DNA fragment spans nucleotides 64 to 618 bp of the *hrcJ* ORF sequence, encoding the 22th-206th amino acids. Used for bacterial two-hybrid assay.
hrcJ-N2F<br>*hrcJ*-N2R	ACAGTTGGATCCCAGCTGTATTCCGGGCTGAC<br>ACAGTTAAGCTT GGCACGCGGCGGCGCCGA	555-bp DNA fragment spans nucleotides 64 to 618 bp of the *hrcJ* ORF sequence, encoding the 22th-206th amino acids. Used for overexpression and pull-down assay.
hpaM-RTP1<br>*hpaM*-RTP2<br>*hpaM*-RTP3<br>*hpaM*-RTP4	AGATAGATGCCCTGGCTGCC<br>CGGTGCGATTGAACGACACG<br>CGGTGCTGGCCACGATCTGCAT<br>ATCGGCCATGCGCAGCACGG	located within the ORF *hapM*, used for 5′-RACE
RP-*hpaM*F<br>RP-*hpaM*R	ACAGTTGAATTCACGCACCGGCCACTTCCCC<br>ACAGTTGGATCCCATGCGGATTCCGGTCAGT	404-bp DNA sequence upstream of the translational start codon of *hpaM* (including ATG). Used for construction of reporter plasmid.
L*avrAC*-F<br>L*avrAC*-R	AAAGGATCC GTTTTTCGATTTCGTGGA<br>AAATCTAGA GGTGCTCATATCCCACAAATTAAGA	577-bp DNA sequence upstream of *avrAC*, used for construction of *avrAC* deletion mutant.
R*avrAC*-F	AAATCTAGATTTGCCGCCTGCCCCGCCCGTTGGTGAA	461-bp DNA sequence downstream of

R*avrAC*-R	CCGAAGCTTATCTCCTGAATCACCACC	*avrAC* , used for construction of *avrAC* deletion mutant.
H*avrAC*-F<br>H*avrAC*-R	AAAGGATCCCCTGGGCAAGGCCAATTATAGCGGCGT<br>AAACTGCAGCTAGTGGTGGTGGTGGTGGTGCTGGTGAACCTGGTT	DNA fragment of 2196-bp *avrAC* promoter sequence and coding sequence fusing with 6×His tag encoded sequence. Used for western blot analysis.
GusF<br>GusR	ACAGTTGGATCCTTACGTCCTGTAGAAACCCC<br>ACAGTTCTGCAGGGCTTTCCCCCCCCCCCCTGCAG	1,832-bp DNA fragment spans nucleotides 4 to 1,835 of the *gusA* ORF.
L3053F<br>L3053R	ACAGTTGGATCCGCAGCGAGGGCCTGGAAG<br>ACAGTTTCTAGAGCGGAAACGGAGCGGGCA	879-bp DNA sequence upstream of *hpaM*$_{\mathrm{Xoc}}$, used for construction of *hpaM*$_{\mathrm{Xoc}}$ deletion mutant.
R3053F<br>R3053R	ACAGTTTCTAGATATCGACCCGCAGCGCTA<br>ACAGTTAAGCTTATTCTGTGGCGTCAGAAG	591-bp DNA sequence downstream of *hpaM*$_{\mathrm{Xoc}}$, used for construction of *hpaM*$_{\mathrm{Xoc}}$ deletion mutant.
C3053F<br>C3053R	ACAGTTGGATCCATGCCTTTGCTTGCCCGCTC<br>ACAGAAGCTTTTATTGCTCGTGGCGCAC	1053-bp DNA fragment of the *hpaM*$_{\mathrm{Xoc}}$ ORF sequence
L01147F<br>L01147R	ACAGTTGGATCCGCAGCGAGGGCATGGAAG<br>ACAGTTTCTAGAGCGGAAACGGAGCGGGCA	879-bp DNA sequence upstream of *hpaM*$_{\mathrm{Xoo}}$, used for construction of *hpaM*$_{\mathrm{Xoo}}$ deletion mutant.
R01147F<br>R01147R	ACAGTTTCTAGATATCGACCCGCAACGCTA<br>ACAGTTAAGCTTATTCTGTGGCGTCAGAAG	591-bp DNA sequence downstream of *hpaM*$_{\mathrm{Xoo}}$, used for construction of *hpaM*$_{\mathrm{Xoo}}$ deletion mutant.
C01147F<br>C01147R	ACAGTTGGATCCATGCCTTTGCTTGCCCGCTC<br>ACAGAAGCTTTTATTGCTCCTGGCGCAC	1053-bp DNA fragment of the *hpaM*$_{\mathrm{Xoo}}$ ORF sequence
01147F<br>01147R	ACAGTTGGATCCATGCCTTTGCTTGCCCGCTC<br>ACAGTTAAGCTTTTAGTGGTGGTGGTGGTGGTGTTGCTCCTGGCGCAC	DNA fragment of 1050-bp *hpaM*$_{\mathrm{Xoo}}$ gene coding sequence of *Xoo* strain fusing with 6×His tag encoding sequence.
3053F<br>3053R	ACAGTTGGATCCATGCCTTTGCTTGCCCGCTC<br>ACAGTTAAGCTTTTAGTGGTGGTGGTGGTGGTGTTGCTCGTGGCGCAC	DNA fragment of 1050-bp *hpaM*$_{\mathrm{Xoc}}$ gene coding sequence of *Xoc* strain fusing with 6×His tag encoding sequence.

[§] The underlined sequences indicate the restriction sites for *Bam*HI, *Eco*RI, *Hin*dIII, *Pst*I, *Xba*I and *Xho*I, respectively.

Table S3. HpaM homologues in *Xanthomonas* spp. and other plant bacterial pathogens

species	gene	Length (aa)	Function predicted	Identity /similarity	Signal peptide	Transmembrane domain	reference
Xanthomonas campestris pv. *raphani*	XCR_1659	349	putative lipoprotein	98.9/99.7	1th-22th aa	8th-29th aa	Bogdanove et al., 2011
Xanthomonas oryzae pv. *oryzicola*	XOC_3053	350	lipoprotein, putative	92/96.9	1th-22th aa	3th-23th aa	Bogdanove et al., 2011
Xanthomonas axonopodis	XAC29_07240	350	hypothetical protein	91.4/96.6	1th-22th aa	3th-23th aa	accession CP004399.1
Xanthomonas oryzae pv. *oryzae*	PXO_01147	350	hypothetical protein	90.9/96.3	1th-22th aa	3th-23th aa	Salzberg et al., 2008
Xanthomonas citri pv. *citri*	XAC1434	348	conserved hypothetical protein	90.6/96.0	1th-20th aa	2th-21th aa	da Silva et al., 2002
Xanthomonas alfalfae	XACM_1423	368	hypothetical protein	87/92.1	1th-40th aa	21th-41th aa	Jalan et al., 2011
Xanthomonas perforans （formerly *Xanthomonas axonopodis* pv. *vesicatoria*）	XPE_3657	368	hypothetical protein	87/92.1	1th-40th aa	21th-41th aa	Potnis et al., 2011
Xanthomonas euvesicatoria (formerly *Xanthomonas campestris* pv. *vesicatoria*)	XCV1491	368	putative secreted protein	87.2/91.8	1th-40th aa	25th-44th aa	Thieme et al., 2005
Xanthomonas fuscans subsp. *fuscans*	XFF4834R_chr30620	368	putative pectin lyase	87.2/91.8	1th-28th aa	21th-44th aa	Darrasse et al., 2013
Xanthomonas translucens pv. *undulosa*	FD63_12180	363	hypothetical protein	62/74.1	1th-41th aa	22th-42th aa	Peng et al., 2016
Xanthomonas sacchari	SB85_05035	348	hypothetical protein	61.1/73.7	1th-16th aa	6th-23th aa	accession CP010409.1
Xanthomonas albilineans	XALc_2014	347	polygalacturonase domain protein	59.9/71.6	1th-23th aa	6th-25th aa	Pieretti et al.,2009
Pseudoxanthomonas spadix	DSC_11120	342	Putative secreted protein	56.4/67.1	1th-17th aa	53th-69th aa	Lee et al., 2012
Xylella fastidiosa subsp. *fastidiosa*	XFLM_05720	342	carbohydrate-binding and sugar hydrolysis	55.1/68.6	1th-24th aa	5th-26th aa	Schreiber et al., 2010
Xylella fastidiosa	XF_0119	342	hypothetical	54.6/67.7	1th-24th aa	7th-28th aa	Simpson et

			protein				al., 2000
Pseudoxanthomonas suwonensis	Psesu_2275	369	OmpA/MotB domain protein	13.4/24.1	1th-22th aa	5th - 23th	accession CP002446.1
Pseudoxanthomonas sp. Root630	No						Bai et al., 2015
Pseudoxanthomonas sp. Root65	No						Bai et al., 2015
Pseudoxanthomonas dokdonensis	No						Patil et al., 2016
Pseudoxanthomonas mexicana	No						GCA_001556105.1
Pantoea ananatis (formerly *Xanthomonas uredovorus*)	No						Hara et al., 2012
Stenotrophomonas maltophilia (formerly *Xanthomonas maltiphilia*)	No						Crossman et al., 2008
Ralstonia solanacearum	No						Salanoubat et al., 2002
Pseudomonas group	No						Stover et al., 2000 Buell et al., 2003
Erwinia group	No						Bell et al., 2004 Smits et al., 2010
Dickeya (formerly *Erwinia chrysanthemi*)	No						Pritchard et al., 2013
Pantoea group	No						Medrano & Bell, 2012 Matsuzawa et al., 2012
Pectobacterium carotovorum	No						Park et al., 2012

Searching the HpaM homologues in plant bacterial pathogens were carried out by blast the genome sequences in KEGG database and NCBI database with the HpaM sequence. All the *Xanthomonas* spp. strains whose genome sequences were available in both databases were scanned and the representative strains were shown here.

Identity/similarity analysis was performed using the Align X program in Vector NTI software. Signal peptide and transmembrane domain predictions were carried with the SMART (Simple Modular Architecture Research Tool) program (http://smart.embl-heidelberg.de) and the TMPRED program (http://www.ch.embnet.org/software/TMPRED_form.html), respectively. **No,** no significant similarity protein (similarity>20%) was found within the genome sequence.

Reference

Bai Y, Müller DB, Srinivas G, Garrido-Oter R, Potthoff E, Rott M, *et al*. (2015) Functional overlap of the *Arabidopsis* leaf and root microbiota. *Nature* **528**: 364-9.

Bell KS, Sebaihia M, Pritchard L, Holden MT, Hyman LJ, Holeva MC, *et al*. (2004) Genome sequence of the enterobacterial phytopathogen *Erwinia carotovora* subsp. *atroseptica* and characterization of virulence factors. *Proc Natl Acad Sci USA*. **101**: 11105-10.

Bogdanove AJ, Koebnik R, Lu H, Furutani A, Angiuoli SV, Patil PB, *et al*. (2011) Two new complete genome sequences offer insight into host and tissue specificity of plant pathogenic *Xanthomonas* spp. *J Bacteriol*. **193**: 5450-5464.

Buell CR, Joardar V, Lindeberg M, Selengut J, Paulsen IT, Gwinn ML, *et al*. (2003) The complete genome sequence of the *Arabidopsis* and tomato pathogen *Pseudomonas syringae* pv. *tomato* DC3000. *Proc Natl Acad Sci USA*. **100**: 10181-6.

Crossman LC, Gould VC, Dow JM, Vernikos GS, Okazaki A, Sebaihia M, *et al*. (2008) The complete genome, comparative and functional analysis of *Stenotrophomonas maltophilia* reveals an organism heavily shielded by drug resistance determinants.

Genome Biol. **9**: R74.

da Silva AC, Ferro JA, Reinach FC, Farah CS, Furlan LR, Quaggio RB, *et al.* (2002) Comparison of the genomes of two *Xanthomonas* pathogens with differing host specificities. *Nature* **417**: 459-63.

Darrasse A, Carrère S, Barbe V, Boureau T, Arrieta-Ortiz ML, Bonneau S, *et al*. (2013) Genome sequence of *Xanthomonas fuscans* subsp. *fuscans* strain 4834-R reveals that flagellar motility is not a general feature of xanthomonads. *BMC Genomics*. **14**: 761.

Hara Y, Kadotani N, Izui H, Katashkina JI, Kuvaeva TM, Andreeva IG, *et al.* (2012) The complete genome sequence of *Pantoea ananatis* AJ13355, an organism with great biotechnological potential. *Appl Microbiol Biotechnol.* **93**: 331-41.

Jalan N, Aritua V, Kumar D, Yu F, Jones JB, Graham JH, *et al*. (2011) Comparative genomic analysis of *Xanthomonas axonopodis* pv. *citrumelo* F1, which causes citrus bacterial spot disease, and related strains provides insights into virulence and host specificity. *J Bacteriol*. **193**: 6342-57.

Lee SH, Jin HM, Lee HJ, Kim JM, and Jeon CO. (2012) Complete genome sequence of the BTEX-degrading bacterium *Pseudoxanthomonas spadix* BD-a59. *J Bacteriol*. **194**: 544.

Matsuzawa T, Mori K, Kadowaki T, Shimada M, Tashiro K, Kuhara S, *et al.* (2012) Genome sequence of *Pantoea agglomerans* strain IG1. *J Bacteriol*. **194**: 1258-9.

Medrano EG and Bell AA. (2012) Genome sequence of *Pantoea* sp. strain Sc 1, an opportunistic cotton pathogen. *J Bacteriol.* **194**: 3019.

Park TH, Choi BS, Choi AY, Choi IY, Heu S, and Park BS. (2012) Genome sequence of *Pectobacterium carotovorum* subsp. *carotovorum* strain PCC21, a pathogen causing soft rot in Chinese cabbage. *J Bacteriol*. **194**: 6345-6.

Patil PP, Midha S, Kumar S, and Patil PB. (2016) Genome Sequence of Type Strains of Genus *Stenotrophomonas*. *Front Microbiol*. **7**: 309.

Peng Z, Hu Y, Xie J, Potnis N, Akhunova A, Jones J, *et al*. (2016) Long read and single molecule DNA sequencing simplifies genome assembly and TAL effector gene analysis of *Xanthomonas translucens*. *BMC Genomics*. **17**: 21.

Pieretti I, Royer M, Barbe V, Carrere S, Koebnik R, Cociancich S, *et al*. (2009) The complete genome sequence of *Xanthomonas albilineans* provides new insights into the reductive genome evolution of the xylem-limited *Xanthomonadaceae*. *BMC Genomics.* **10**: 616.

Potnis N, Krasileva K, Chow V, Almeida NF, Patil PB, Ryan RP, *et al*. (2011) Comparative genomics reveals diversity among *xanthomonads* infecting tomato and pepper. *BMC Genomics*. **12**: 146.

Pritchard L, Humphris S, Saddler GS, Elphinstone JG, Pirhonen M, and Toth IK. (2013) Draft genome sequences of 17 isolates of the plant pathogenic bacterium *dickeya*. *Genome Announc*. **1**(6).

Salanoubat M, Genin S, Artiguenave F, Gouzy J, Mangenot S, Arlat M, *et al.* (2002) Genome sequence of the plant pathogen *Ralstonia solanacearum*. *Nature* **415**: 497-502.

Salzberg SL, Sommer DD, Schatz MC, Phillippy AM, Rabinowicz PD, Tsuge S, et al. (2008) Genome sequence and rapid evolution of the rice pathogen *Xanthomonas oryzae* pv. *oryzae* PXO99^{A}. *BMC Genomics*. **9**: 204.

Schreiber HL, Koirala M, Lara A, Ojeda M, Dowd SE, Bextine B, *et al*. (2010) Unraveling the first *Xylella fastidiosa* subsp. *fastidiosa* genome from Texas. *Southwestern Entomologist.* **35**: 479-483.

Simpson AJ, Reinach FC, Arruda P, Abreu FA, Acencio M, Alvarenga R, *et al.* (2000)

The genome sequence of the plant pathogen *Xylella fastidiosa*. The *Xylella fastidiosa* Consortium of the Organization for Nucleotide Sequencing and Analysis. *Nature* **406**: 151-9.

Smits TH, Rezzonico F, Kamber T, Blom J, Goesmann A, Frey JE, *et al*. (2010) Complete genome sequence of the fire blight pathogen Erwinia amylovora CFBP 1430 and comparison to other *Erwinia* spp. *Mol Plant Microbe Interact*. **23**: 384-93.

Stover CK, Pham XQ, Erwin AL, Mizoguchi SD, Warrener P, Hickey MJ, *et al*. (2000) Complete genome sequence of *Pseudomonas aeruginosa* PAO1, an opportunistic pathogen. *Nature* **406**: 959-64.

Thieme F, Koebnik R, Bekel T, Berger C, Boch J, Büttner D, *et al*. (2005) Insights into genome plasticity and pathogenicity of the plant pathogenic bacterium *Xanthomonas campestris* pv. *vesicatoria* revealed by the complete genome sequence. *J Bacteriol*. **187**: 7254-66.
